# Potent high-avidity neutralizing antibodies and T cell responses after COVID-19 vaccination in individuals with B cell lymphoma and multiple myeloma

**DOI:** 10.1038/s43018-022-00502-x

**Published:** 2022-12-21

**Authors:** Andrea Keppler-Hafkemeyer, Christine Greil, Paul R. Wratil, Khalid Shoumariyeh, Marcel Stern, Annika Hafkemeyer, Driti Ashok, Alexandra Hollaus, Gaia Lupoli, Alina Priller, Marie L. Bischof, Gabriele Ihorst, Monika Engelhardt, Reinhard Marks, Jürgen Finke, Hannah Bertrand, Christopher Dächert, Maximilian Muenchhoff, Irina Badell, Florian Emmerich, Hridi Halder, Patricia M. Spaeth, Percy A. Knolle, Ulrike Protzer, Michael von Bergwelt-Baildon, Justus Duyster, Tanja N. Hartmann, Andreas Moosmann, Oliver T. Keppler

**Affiliations:** 1 Department of Medicine I, Medical Center-University of Freiburg, Faculty of Medicine, University of Freiburg, Freiburg, Germany; 2 Max von Pettenkofer Institute and Gene Center, Virology, National Reference Center for Retroviruses, LMU München, Munich, Germany; 3 German Center for Infection Research (DZIF), Munich Partner Site, Munich, Germany; 4 German Cancer Consortium (DKTK), partner site Freiburg, and German Cancer Research Center (DKFZ), Heidelberg, Germany; 5 Medizinische Klinik und Poliklinik III, LMU Klinikum, LMU München, Munich, Germany; 6 Institute of Molecular Immunology and Experimental Oncology, University Hospital rechts der Isar, Technical University of Munich (TUM) School of Medicine, Munich, Germany; 7 Clinical Trials Unit, Faculty of Medicine and Medical Center, University of Freiburg, Freiburg, Germany; 8 Institute for Transfusion Medicine and Gene Therapy, Freiburg University Medical Center, Faculty of Medicine, University of Freiburg, Freiburg, Germany; 9 Helmholtz Munich, Munich, Germany; 10 Institute of Virology, Technical University of Munich School of Medicine/Helmholtz Munich, Munich, Germany

**Keywords:** SARS-CoV-2, Viral infection, Cancer

## Abstract

Individuals with hematologic malignancies are at increased risk for severe coronavirus disease 2019 (COVID-19), yet profound analyses of COVID-19 vaccine-induced immunity are scarce. Here we present an observational study with expanded methodological analysis of a longitudinal, primarily BNT162b2 mRNA-vaccinated cohort of 60 infection-naive individuals with B cell lymphomas and multiple myeloma. We show that many of these individuals, despite markedly lower anti-spike IgG titers, rapidly develop potent infection neutralization capacities against several severe acute respiratory syndrome coronavirus 2 variants of concern (VoCs). The observed increased neutralization capacity per anti-spike antibody unit was paralleled by an early step increase in antibody avidity between the second and third vaccination. All individuals with hematologic malignancies, including those depleted of B cells and individuals with multiple myeloma, exhibited a robust T cell response to peptides derived from the spike protein of VoCs Delta and Omicron (BA.1). Consistently, breakthrough infections were mainly of mild to moderate severity. We conclude that COVID-19 vaccination can induce broad antiviral immunity including ultrapotent neutralizing antibodies with high avidity in different hematologic malignancies.

## Abstract (web summary)

Keppler and colleagues show that individuals with hematological cancers rapidly develop potent infection-neutralizing antibodies and a robust T cell response against several SARS-CoV-2 variants of concern in response to mRNA vaccination.

## Main

Coronavirus disease 2019 (COVID-19) results in increased morbidity and mortality in individuals with cancer^1–6^. Hematologic malignancies are frequently associated with secondary immunodeficiency, and affected individuals have a higher risk of experiencing severe COVID-19 than individuals with solid cancer, with reported odds ratios of 1.6 to 3.3 (refs. 1,2). In a group of individuals with hematologic malignancies, those who had recent chemotherapy were at increased risk of death during COVID-19-associated hospital admission, with an odds ratio of 2.09 (ref. 1).

Authorized vaccines against severe acute respiratory syndrome coronavirus 2 (SARS-CoV-2) are effective in preventing and mitigating the course of COVID-19 and in partially reducing viral transmission in immunocompetent individuals, inducing both robust humoral and T cell responses^7–10^. Recent data indicate that the presence of both binding and neutralizing antibodies may be predictive of protection against symptomatic disease^11^. A high-throughput neutralization assay using authentic, replication-competent viruses allowed us to gain deeper insight into humoral immunity against variants of concern (VoCs) in a longitudinal cohort of healthy individuals^12^. In this study, three timely spaced exposures to the spike protein of SARS-CoV-2 from either vaccination or infection resulted in increases in neutralization capacity per anti-spike antibody unit and increases in antibody avidity^12^. This suggested that the quality rather than the mere quantity of anti-spike IgG may be critical for predicting the humoral vaccine response and possibly also the protection against symptomatic disease. In particular, the maturation of antibodies to the spike protein may play an important role in the development of potent neutralizing responses^12,13^. Interestingly, in individuals who recovered from recurrent COVID-19, the presence of low-avidity IgG molecules targeting the receptor-binding domain of the SARS-CoV-2 spike protein during reinfection was shown to be a negative prognostic factor for developing severe COVID-19 (ref. 14). The development of cellular immunity following COVID-19 vaccination in individuals with cancer has been addressed in recent studies^2,15–20^; however, these studies often lack prevaccination samples or fail to assess specific or cross-reactive T cell responses to seasonal human β-coronaviruses (HCoVs) or more recent variants of SARS-CoV-2.

Highlighted in a recent review^21^, considerable uncertainty still remains regarding the efficacy of COVID-19 vaccination in individuals with cancer. Laboratory-based studies on individuals with hematologic malignancies have started to focus on the assessment of longitudinal immune responses induced by COVID-19 vaccines in subgroups with different B cell lymphomas (LY), multiple myeloma (MM) and their related treatment regimens^17,22–25^. These reports have provided more differentiated insight yet have also highlighted the challenges of analyses of COVID-19 vaccine responses in these diverse groups of individuals with hematologic cancers. Humoral vaccine responses in individuals with cancer are frequently still explored solely by determining the quantity of anti-spike IgG^26–32^ or by the use of surrogate neutralization assays^33–37^ that may not faithfully reflect results from live virus neutralization assays, which are considered the gold standard for sensitive quantification of functional, infection-blocking antibodies with in vivo relevance^38,39^. The detailed assessment of COVID-19 vaccine responses in immunocompromised individuals is also important to define the clinical need for early pharmacological interventions with oral antivirals, including paxlovid or molnupiravir^40^, or the potential use of neutralizing monoclonal antibodies^41–45^.

In the current study, we examined the dynamics of humoral and cellular immune responses in a longitudinal cohort of 60 individuals with either LY or MM (vaccinated primarily with BNT162b2 mRNA) by quantifying antibodies to the SARS-CoV-2 spike protein, antibody avidity and neutralization capacity in serum. Moreover, we performed an enzyme-linked immunospot (ELISpot) assay stimulating interferon-γ (IFNγ) release by peptide pools of SARS-CoV-2 spike S1 and S2 domains of Delta and partially of Omicron (BA.1) to quantitatively assess the SARS-CoV-2- and seasonal HCoV OC43-directed T cell responses to COVID-19 in 53 of these individuals with cancer.

## Results

### Cohort characteristics and study design

**Fig. 1:**
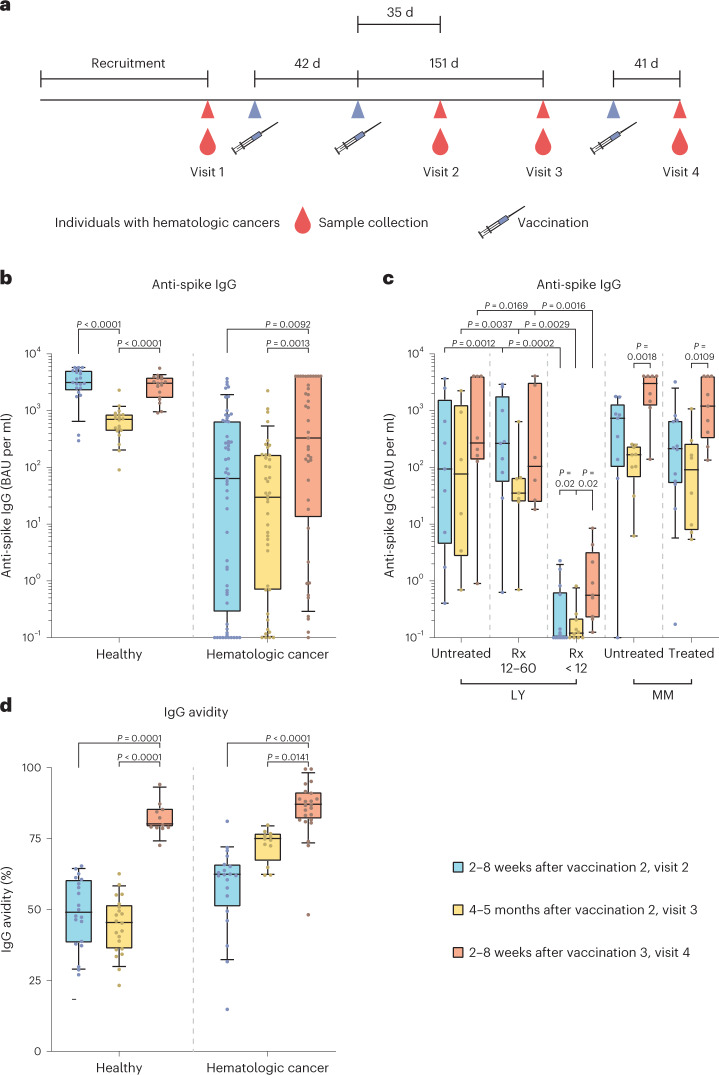
Study time chart, anti-spike IgG levels and IgG avidity in individuals with hematologic neoplasia and in healthy individuals at different time points after COVID-19 vaccination. **a**, Time chart of the study depicting time points of vaccination and blood sample collection in a cohort of individuals with hematologic cancers. Prevaccination samples were collected shortly before vaccination 1 (visit 1). Visit 2 occurred 2–8 weeks (median of 35 d) after vaccination 2. Visit 3 occurred 4–5 months (median of 149 d) after vaccination 2. Visit 4 occurred 2–8 weeks (median of 40 d) after vaccination 3. Vaccinations 1 and 2 were administered 6 weeks apart (median of 42 d); vaccinations 2 and 3 were 6 months apart (median of 189 d). **b**–**d**, Data are depicted as box plots with median, bounds between upper and lower quartiles and whiskers between the 10th and 90th percentiles. Differences between time points (visit 2, blue; visit 3, yellow; visit 4, red) were analyzed for statistical significance using the Kruskal–Wallis test with Dunn’s multiple-testing correction. Brackets show statistically significant differences, and precise numerical *P* values are indicated. Absence of brackets indicates absence of significance. **b**, Anti-spike S1 domain IgG titers in BAU per ml at different time points after vaccinations 2 and 3. The following samples were analyzed for groups of healthy individuals and individuals with hematologic neoplasia: 2–8 weeks after vaccination 2 (visit 2, blue; *N* = 21/*N* = 57), 4–5 months after vaccination 2 (visit 3, yellow; *N* = 20/*N* = 42) and 2–8 weeks after vaccination 3 (visit 4, red; *N* = 19/*N* = 42). **c**, Levels of antibody specific to the spike S1 domain after vaccinations 2 and 3 comparing subgroups of individuals with hematologic neoplasia; untreated LY (visit 2, *N* = 9; visit 3, *N* = 6; visit 4, *N* = 8)/LYs treated with Rx 12–60 months before receiving the first vaccination (Rx 12–60; visit 2, *N* = 9; visit 3, *N* = 7; visit 4, *N* = 6)/LYs treated with Rx in the last 12 months before the first vaccination (Rx < 12; visit 2, *N* = 14; visit 3, *N* = 10; visit 4, *N* = 9)/untreated MM (visit 2, *N* = 9; visit 3, *N* = 9; visit 4, *N* = 8)/treated MM (visit 2, *N* = 12; visit 3, *N* = 8; visit 4, *N* = 9). **d**, Avidity of anti-spike IgG at different time points after vaccinations 2 and 3; healthy individuals: visit 2 (*N* = 20)/visit 3 (*N* = 21)/visit 4 (*N* = 11); individuals with hematologic malignancies: visit 2 (*N* = 20)/visit 3 (*N* = 12)/visit 4 (*N* = 23). Source data

We characterized the SARS-CoV-2-specific humoral and cellular immune responses after two and three vaccinations with mostly BNT162b2 mRNA in 60 individuals with various LYs and MM. Individuals were treated at the Freiburg University Medical Center, Germany (baseline participant characteristics are in Table 1) and were followed up from before their initial COVID-19 vaccination (mostly in March or April 2021) to January 2022, the latter time point, on average, 41 d after the third vaccination (booster). Of note, regardless of the subgroup, the remission state was ‘stable disease’ or better in almost all individuals at the time of first vaccination. Only two individuals diagnosed with MM showed ‘active disease’ (initial diagnosis or progressive disease, respectively). The remission state of each individual in the LY subgroup treated with the monoclonal antibody (mAb) to CD20 rituximab (Rx) less than 12 months before the first vaccination (LY Rx < 12) and the time between last Rx infusion and the respective COVID-19 vaccinations 1–3 are shown in Extended Data Table 1. The time chart of vaccinations 1–3 and the time points of sample collection (visits 1–4) are shown in Fig. 1a. Data on the individuals with cancer were compared to data of an age- and sex-matched cohort of healthy healthcare workers, reported recently for humoral responses^12^, and were included in the quantification of T cell responses in the current study.

**Table 1:** Baseline characteristics of vaccinated individuals with hematologic malignancies

Cohort characteristics, *N* = 60^a^	*N* (%)
Age, median (IQR), years	63.5 (58–70.25)
Male	33 (55)
Ethnicity, white	60 (100)
SARS-CoV-2 infection or detection of nucleocapsid antibody	
Detection of nucleocapsid antibody and/or previous SARS-CoV-2 infection before first vaccination, respectively	0 (0)
Detection of nucleocapsid antibody between first and second vaccination	1 (2)^b^
Detection of nucleocapsid antibody after second vaccination	5 (8)^b^
Detection of nucleocapsid antibody after third vaccination	2 (3)^b^
First COVID-19 vaccination	
BNT162b2 mRNA vaccine (Comirnaty, BioNTech/Pfizer)	51 (85)
mRNA-1273 vaccine (Spikevax, Moderna)	1 (2)
ChAdOx1 nCoV-19 vaccine (AZD1222, Vaxzevria, Oxford/AstraZeneca)	8 (13)
Second COVID-19 vaccination	
BNT162b2 mRNA vaccine (Comirnaty, BioNTech/Pfizer)	58 (97)
ChAdOx1 nCoV-19 vaccine (AZD1222, Vaxzevria, Oxford/AstraZeneca)	2 (3)^c^
Third COVID-19 vaccination	
BNT162b2 mRNA vaccine (Comirnaty, BioNTech/Pfizer)	36 (77)
mRNA-1273 vaccine (Spikevax, Moderna)	11 (23)
Time from first blood draw to first vaccination, median (IQR) (d)	2 (1–6)
Time from first to second vaccination, median (IQR) (d)	42 (24–42)
Time from second vaccination to second blood draw, median (IQR) (d)	35 (30–41)
Time from second vaccination to third blood draw, median (IQR) (d)	151 (137–164)
Time from second to third vaccination, median (IQR) (d)	189 (174–207)
Time from third vaccination to fourth blood draw, median (IQR) (d)	41 (31–56)
Oncological history	
Hematologic malignancies, *N* = 60	
Diagnosis	
LY	38 (63)
FL	12 (20)
MCL	4 (7)
MZL	4 (7)
CLL	12 (20)
MALT	2 (3)
DLBCL	3 (5)
Waldenström (Myd88 positive)	1 (2)
Anticancer treatment	
Currently receiving anti-CD20 therapy or <12 months prior	14 (23)
mAb to CD20 therapy 12–60 months prior	10 (17)
mAb to CD20 therapy >60 months prior or treatment naive	10 (17)
Venetoclax	1 (2)
Ibrutinib	3 (5)
Myeloma	22 (37)
MM	19 (32)
SMM	2 (3)
MGUS	1 (2)
Anticancer treatment	
Treatment naive (‘untreated’)	5 (8)
Lenalidomide 25–74 months (median 33 months) prior or autologous stem cell transplantation longer, on average, than 76 months before vaccination 1 (considered ‘untreated’)	5 (8)
Currently receiving lenalidomide (as maintenance or relapse therapy) and 25 months (median) after autologous stem cell transplantation (considered ‘treated’)	7 (12)
Currently receiving targeted therapy (considered ‘treated’)	5 (8)
Previous autologous stem cell transplant	18 (30)

Demographic, epidemiological and clinical data (for example, cancer type and treatment history) of 60 individuals enrolled in the trial.

At visit 1, the hematologic cohort included 22 individuals with MM (*N* = 10 untreated MM; *N* = 12 treated MM) and 38 individuals with LY (*N* = 10 untreated LY; *N* = 14 LY treated with Rx for <12 months; *N* = 10 LY treated with Rx for 12–60 months before vaccination; *N* = 4 LY treated with venetoclax or ibrutinib).

‘Untreated’ MM was defined as either treatment naive or end of lenalidomide treatment 25 to 74 (median of 33) months before vaccination 1 and/or autologous stem cell transplantation longer than 59 to 90 (median of 76) months prior. ‘Treated’ MM was defined as ongoing treatment of individuals with mainly lenalidomide as maintenance or relapse therapy or any other targeted, myeloma-specific therapy. All treated individuals had had autologous stem cell transplantation 3–66 (median of 25) months prior.

The majority of individuals (87%) had received a homologous vaccination with BNT162b mRNA; six (10%) had received a heterologous vaccination with the viral vector-based vaccine AZD1222 followed by BNT162b mRNA.

For booster vaccination, all individuals received an mRNA-based vaccine.

FL, follicular lymphoma; MCL, mantle cell lymphoma; MZL, marginal zone lymphoma; CLL, chronic lymphocytic leukemia; MALT, mucosa-associated lymphoid tissue lymphoma; DLBCL, diffuse large B cell lymphoma; SMM, smoldering multiple myeloma; MGUS monoclonal gammopathy of undetermined significance

^a^Neither Evusheld nor any other anti-spike neutralizing antibody approved in Germany was given to participants in our cohort in a prophylactic setting.

^b^Of note, all serum samples were characterized for anti-nucleocapsid titers. In case of a positive anti-nucleocapsid titer, participants were excluded from further statistical analysis.

^c^The two individuals vaccinated with the AZD1222 vaccine twice were excluded from further statistical analyses.

### Longitudinal dynamics of anti-SARS-CoV-2 spike IgG

**Extended Data Fig. 1:**
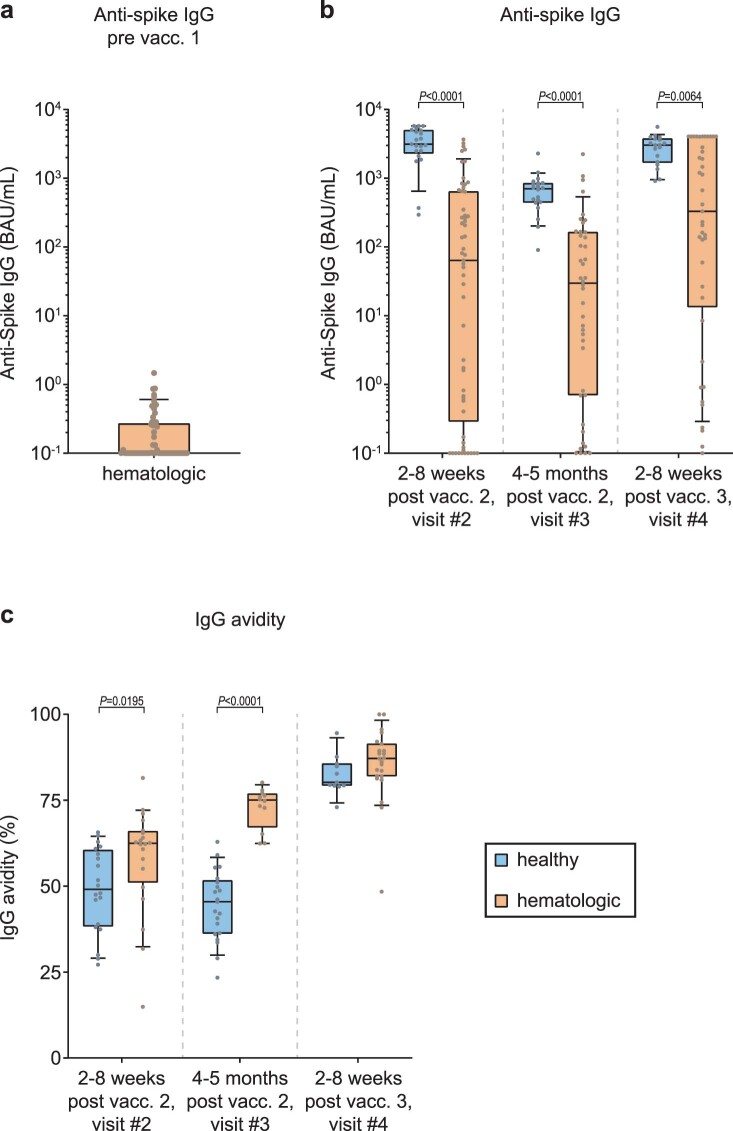
Comparison of IgG-type antibody levels and avidity in healthy individuals and patients with hematologic neoplasia at different time points before and after COVID-19 vaccination. Data is depicted as boxplots with median, bounds between upper and lower quartiles, and whiskers between the 10th and 90th percentile. Differences between groups (healthy individuals/ patients with hematologic malignancies) were analyzed for statistical significance using the Mann-Whitney test. Brackets show statistically significant differences. Absence of brackets or p-values indicate absence of significance. **a**, Anti-spike S1 domain IgG titers in BAU/mL prior to vaccination in patients with hematologic malignancies (n = 56). **b**, Anti-spike S1 domain IgG antibody levels at different time points after COVID-19 vaccinations #2 and #3 in healthy individuals (visit #2/ visit# 3/ visit #4: n = 21/20/19, respectively) and in cancer patients (visit #2/ visit# 3/ visit #4: n = 57/42/42, respectively). **c**, Anti-spike IgG antibody avidity at different time points after vaccinations #2 and #3. Healthy individuals: visit #2 (n = 20)/ visit #3 (n = 21)/ visit #4 (n = 11); Hematologic malignancies: visit #2 (n = 20/ visit #3 (n = 12)/ visit #4 (n = 23). Source data

We first quantified vaccine-induced spike S1 domain-reactive antibodies before vaccination 1 (visit 1), at 2–8 weeks (visit 2) or 4–5 months (visit 3) after vaccination 2 and at 2–8 weeks after vaccination 3 (booster shot; visit 4) in infection-naive individuals with cancer. Individuals testing positive for nucleocapsid antibodies were excluded (Table 1). Baseline spike-specific antibody levels were low or undetectable (Extended Data Fig. 1a). Early after the second vaccination (visit 2), the median IgG titer of individuals with hematologic malignancies was 49.1-fold lower than that of healthy individuals (Extended Data Fig. 1b). At visit 3, a 23.6-fold difference was still observed between these two groups (Extended Data Fig. 1b). Notably, at visit 4, the factor of difference decreased to 9.2-fold (Extended Data Fig. 1b). A considerable interparticipant variability was observed, likely due to different Rx treatment regimens; at 2-8 weeks after vaccination 2, individuals with untreated LY and individuals with a longer interval since the last administration of Rx (Rx 12–60 months) showed reduced vaccine-induced median IgG levels that were 33-fold and 11.9-fold lower, respectively, than in matched healthy individuals (Fig. 1b,c). However, the 14 Rx < 12 individuals with LY showed low or negative anti-spike IgG titers after both the second and third vaccinations, consistent with recent studies^24,28,46^, whereas untreated individuals with LY mostly presented with measurable anti-spike IgG titers (Fig. 1c). A trend toward lower median anti-spike IgG concentrations was observed at visit 3 than at visit 2 in both healthy individuals (4.6-fold reduction) and individuals with hematologic malignancies (2.1-fold reduction; Fig. 1b).

Anti-spike IgG titers at visit 4 were markedly elevated in both groups compared to at visit 3 (Fig. 1b). Individuals with hematologic malignancies showed a 9.3-fold rise at visit 4 compared to titers observed at visit 3, and healthy individuals displayed a 4.9-fold increase. At visit 4, median anti-spike IgG concentrations in healthy individuals were 10.8-fold higher than in individuals with cancer (Extended Data Fig. 1b). In individuals with MM, the dynamics of vaccine-induced anti-spike IgG responses were comparable to those observed in healthy individuals, with a marked increase of anti-spike IgG titers from visit 3 to visit 4 (Fig. 1b,c). In these individuals, ongoing therapy had no significant influence on anti-spike IgG levels (Fig. 1c). Among the few individuals with chronic lymphocytic leukemia treated with novel agents, such as the Bruton’s tyrosine kinase (BTK) inhibitor ibrutinib (*N* = 3) or the B cell leukemia/lymphoma-2 (BCL-2) inhibitor venetoclax (*N* = 1), only one individual, an individual treated with ibrutinib, had a vaccine-induced anti-spike IgG response above the cutoff of 10 binding antibody units (BAU) per ml.

Taken together, 2–8 weeks after vaccination 2, 22 individuals with hematologic cancer showed negative or low-level anti-spike IgG levels, corresponding to 39% of the 57 individuals included in the statistical analysis, whereas all 25 matched healthy individuals displayed robust anti-spike IgG responses. None of the individuals with an Rx < 12 regimen developed an anti-spike IgG response markedly above background. Of the 35 individuals with hematologic cancer with detectable anti-spike IgG concentrations at visit 2, all still had a positive IgG response at visit 3 and a rise in antibody titers after booster vaccination.

### Early increase in avidity of anti-spike IgG in individuals with hematologic cancer

Recently, we reported a maturation of anti-spike IgG avidity in healthy individuals over time and after each encounter with the SARS-CoV-2 spike protein (vaccine induced or infection related), especially after a third exposure to the spike protein^12^. Here, we quantified the avidity of serum IgGs binding to the SARS-CoV-2 spike 1 (S1) and spike 2 (S2) ectodomains. For individuals with hematologic malignancies, we observed a 20% increase in antibody avidity from visit 2 to visit 3, a significant increase of 16% from visit 3 to visit 4 and a highly significant increase from visit 2 to visit 4 (Fig. 1d). Comparing the two cohorts, we noted that individuals with hematologic malignancies showed significantly higher antibody avidities at visit 2 than healthy individuals at 14 d and even 103 d after vaccination 2. Moreover, individuals with cancer showed a drastically higher anti-spike IgG avidity at visit 3 than healthy individuals (Extended Data Fig. 1c), while avidity levels were comparable in both study groups at visit 4 (Fig. 1d and Extended Data Fig. 1c). In sum, our findings unexpectedly reveal that in those vaccinated individuals with hematologic cancer who were able to mount a humoral response, the avidity of anti-spike IgG rose rapidly after the second vaccination and was markedly higher after the second and before the third vaccination than in healthy individuals, contrasting the concurrently reduced anti-spike IgG titers in individuals with cancer.

### Broad infection-neutralizing activity after COVID-19 vaccination

Next, we quantified infection-neutralizing antibodies in sera collected before and longitudinally after COVID-19 vaccination (Fig. 1a) from individuals with cancer and compared these results to those from matched healthy individuals, reported recently^12^. We used a live virus neutralization assay against six SARS-CoV-2 variants, reflecting the evolutionary course of SARS-CoV-2 during the first 2 years of the pandemic. We assessed the serum neutralization titers by measuring half-maximal inhibitory concentrations (IC_50_) for preventing virus-mediated cytotoxicity^12,47^.

**Fig. 2:**
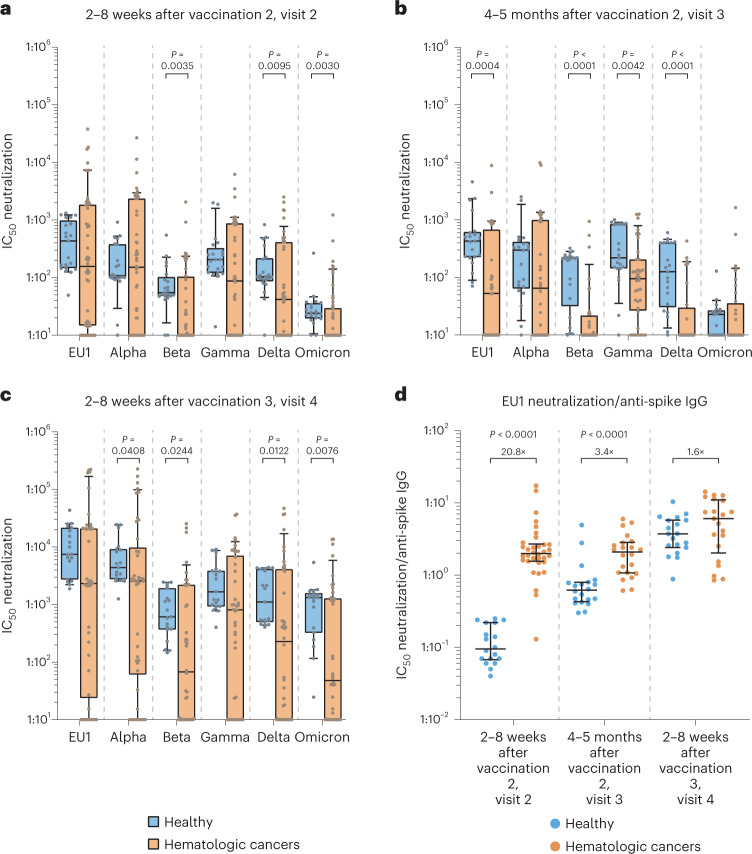
Comparison of infection neutralization activities for SARS-CoV-2 VoCs in individuals with hematologic neoplasia and healthy individuals at different time points after COVID-19 vaccination. Serum dilutions for half-maximal infection neutralization capacities normalized to 10^7^ viral RNA copies (neutralization IC_50_ values) are depicted for different SARS-CoV-2 variants as box plots with median, bounds between upper and lower quartiles and whiskers between the 10th and 90th percentiles. Differences between groups were tested for their statistical significance using the Mann–Whitney test. Brackets show statistically significant differences, and precise numerical *P* values are indicated. Absence of brackets or *P* values indicates absence of significance. **a**, Neutralization IC_50_ values at 2–8 weeks after vaccination 2 (visit 2) for SARS-CoV-2 variants in healthy individuals (*N* = 21) versus in individuals with hematologic malignancies (*N* = 56). **b**, Neutralization IC_50_ values at 4–5 months after vaccination 2 (visit 3) for SARS-CoV-2 variants (EU1, Alpha, Beta, Gamma, Delta and Omicron BA.1, respectively) in healthy individuals (*N* = 21, 21, 21, 21, 21 and 21, respectively) versus in individuals with hematologic malignancies (*N* = 36, 36, 36, 36, 36 and 36, respectively). **c**, Neutralization IC_50_ values at 2–8 weeks after vaccination 3 (visit 4) for SARS-CoV-2 variants (EU1, Alpha, Beta, Gamma, Delta and Omicron BA.1, respectively) in healthy individuals (*N* = 19, 19, 19, 19, 19 and 19, respectively) versus in individuals with hematologic malignancies (*N* = 42, 42, 42, 42, 42 and 42, respectively). **d**, Ratios between infection neutralization IC_50_ values for EU1 values and anti-spike S1 domain titers at visits 2, 3 and 4, respectively, comparing vaccinated healthy individuals (*N* = 18, 20 and 19, respectively) and individuals with hematologic malignancies (*N* = 34, 22 and 21, respectively). Medians and IQRs (error bars) are depicted. Source data

**Extended Data Fig. 2:**
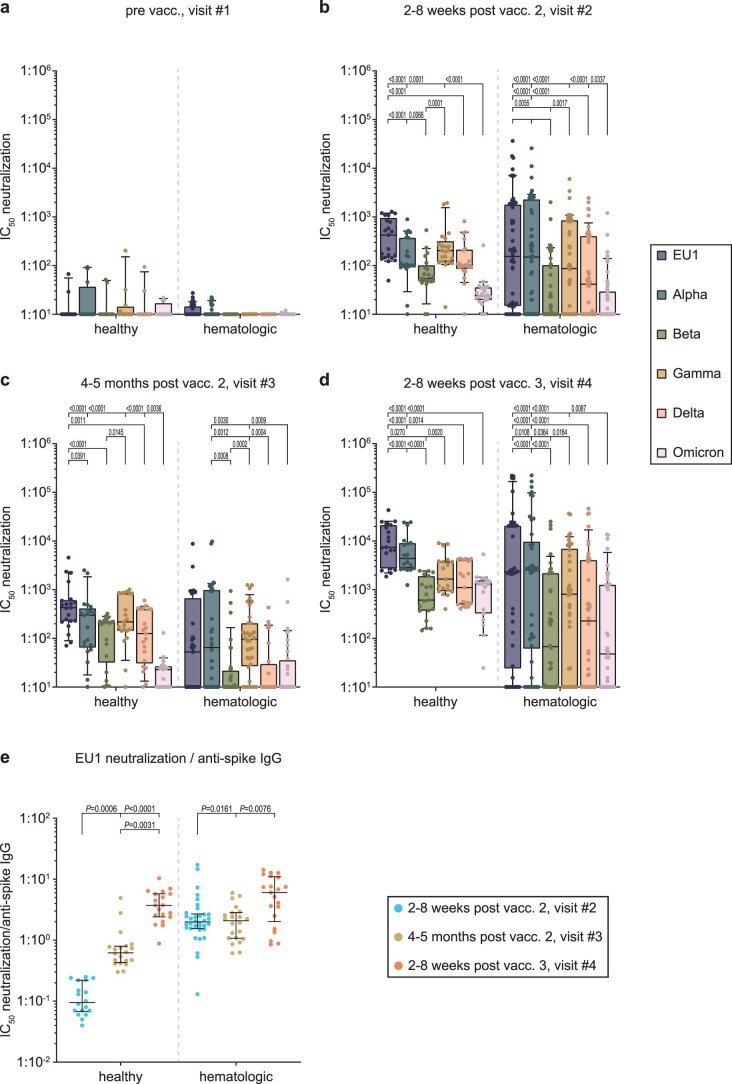
Variant-centered comparison of infection-neutralization activities against SARS-CoV-2 in healthy individuals and patients with hematologic neoplasia at different time points before and after vaccination, and longitudinal evaluation of ratios between infection-neutralization and anti-spike antibody levels. Serum dilutions for half-maximal infection-neutralization capacities normalized to 10^7^ viral RNA copies (neutralization IC_50_-values) are depicted for different SARS-CoV-2 variants as box plots with median, bounds between upper and lower quartiles, and whiskers between the 10th and 90th percentiles. **a**, Neutralization IC_50_-values pre-vaccination (visit #1) from healthy individuals (n = 12) and from patients with hematologic neoplasia (n = 53) were analyzed. **b**, Neutralization IC_50_-values 2-8 weeks post vaccination #2 (visit #2) from healthy individuals (n = 21) and patients with hematologic neoplasia (n = 56) were analyzed. **c**, Neutralization IC_50_-values 4-5 months post vaccination #2 (visit #3) from healthy individuals (n = 21) and from patients with hematologic neoplasia (n = 36) were analyzed. **d**, Neutralization IC_50_-values 2-8 weeks post vaccination #3 (visit #4) from healthy individuals (n = 19) and from patients with hematologic neoplasia (n = 42) were analyzed. **e**, Longitudinal evaluation of ratios between infection-neutralization IC_50_ values for EU1 and anti-spike S1 domain antibody titers for EU1 in vaccinated healthy individuals (visits #2, #3 and #4: n = 18/20/19, respectively) and hematologic patients (visits #2, #3 and #4: n = 34/22/21, respectively). Medians and interquartile ranges (error bars) are depicted. Differences between groups were tested for their statistical significance using the two-tailed Friedman test with Dunn’s multiple testing correction in (**a**-**d**), and the Kruskal-Wallis-test with Dunn’s multiple testing correction in (**e**). Brackets show statistically significant differences. Absence of brackets or p-values indicates absence of significance. Source data

The prevaccination baseline neutralization activity is depicted in Extended Data Fig. 2a. The dynamics of a SARS-CoV-2 variant-centered comparison of neutralization activities in longitudinal serum specimens are shown in Fig. 2a–c. In individuals with cancer and healthy individuals, the infection neutralization capacities for VoC Omicron (BA.1) and, albeit less pronounced, for VoCs Beta and Delta were mostly lower than for the other SARS-CoV-2 variants at all time points investigated (Extended Data Fig. 2b-d), confirming their immune escape properties^48–50^.

At visit 2, sera from our cohort had lower neutralization activity against the different SARS-CoV-2 variants than sera from healthy individuals by, on average, 2.3- to 5.4-fold, reaching statistical significance for VoCs Beta, Delta and Omicron (Fig. 2a). At visit 3, the cancer cohort showed a 2.3- to 21.3-fold reduced neutralization capacity, which was statistically significant for VoCs Beta and Delta (Fig. 2b), EU1 (Fig. 2b) and Gamma (Fig. 2b) but not for VoCs Alpha and Omicron. After the booster vaccination, individuals with hematologic malignancies displayed a moderately reduced level of neutralization activity for VoCs Omicron and Beta compared to healthy individuals (that is, by 27.6- and 9.1-fold, respectively), whereas neutralization differences for the other VoCs were less pronounced (1.7- to 4.9-fold; Fig. 2c).

Next, we determined the ratio between neutralization IC_50_ values for EU1 and anti-spike IgG concentrations to obtain insight into the relative efficacy of spike-targeting antibodies in serum for live virus neutralization. Most remarkably, shortly after the second vaccination, anti-spike IgG-positive individuals with cancer displayed a 20.8-fold higher neutralization capacity per BAU than healthy individuals (Fig. 2d). Over time, the most pronounced increase in neutralization capacity of antibodies to SARS-CoV-2 was observed in healthy donors (Extended Data Fig. 2e). Notably, the neutralization potency per anti-spike IgG in individuals with hematologic malignancies at day 35 after the second vaccination was significantly higher than at day 103 and even day 210 in the group of healthy individuals. In line with this finding, this ratio was significantly lower in healthy individuals 5 months after the second vaccination (visit 3) than in individuals with hematologic malignancies and reached comparable levels only after the third vaccination (Fig. 2d).

**Fig. 3:**
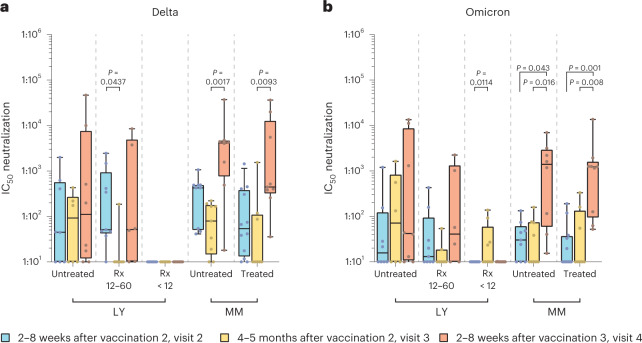
Longitudinal comparison of infection neutralization activities against Delta and Omicron in sera from subgroups of individuals with hematologic neoplasia. **a**, Infection neutralization IC_50_ values for the Delta VoC. **b**, Infection neutralization IC_50_ values for the Omicron (BA.1) VoC. Neutralization IC_50_ values in serum are depicted for different SARS-CoV-2 variants as box plots with median, bounds between upper and lower quartiles and whiskers between the 10th and 90th percentiles. Differences between time points were tested for their statistical significance using the Kruskal–Wallis test with Dunn’s multiple-testing correction. Brackets show statistically significant differences, and precise numerical *P* values are indicated. Absence of brackets or *P* values indicates absence of statistical significance. Sera from the following subgroups of individuals were analyzed at visit 2 (blue), visit 3 (yellow) and visit 4 (red); untreated LY (Delta VoC: visit 2/visit 3/visit 4: *N* = 8/6/8, respectively; Omicron (BA.1) VoC: visit 2/visit 3/visit 4: *N* = 8/6/8, respectively); individuals with LY treated with Rx 12–60 months before receiving the first vaccination (Rx 12–60; Delta: visit 2/visit 3/visit 4: *N* = 9/7/6, respectively; Omicron (BA.1): visit 2/visit 3/visit 4: *N* = 9/7/6, respectively); individuals with LY treated with Rx in the last 12 months before the first vaccination (Rx < 12; Delta: visit 2/visit 3/visit 4: *N* = 14/9/10, respectively; Omicron (BA.1): visit 2/visit 3/visit 4: *N* = 14/9/9, respectively); untreated MM (Delta: visit 2/visit 3/visit 4: *N* = 9/9/8, respectively; Omicron (BA.1): visit 2/visit 3/visit 4: *N* = 9/8/8, respectively); treated MM (Delta: visit 2/visit 3/visit 4: *N* = 12/8/9, respectively; Omicron (BA.1): visit 2/visit 3/visit 4: *N* = 12/8/9, respectively). Source data

**Extended Data Fig. 3:**
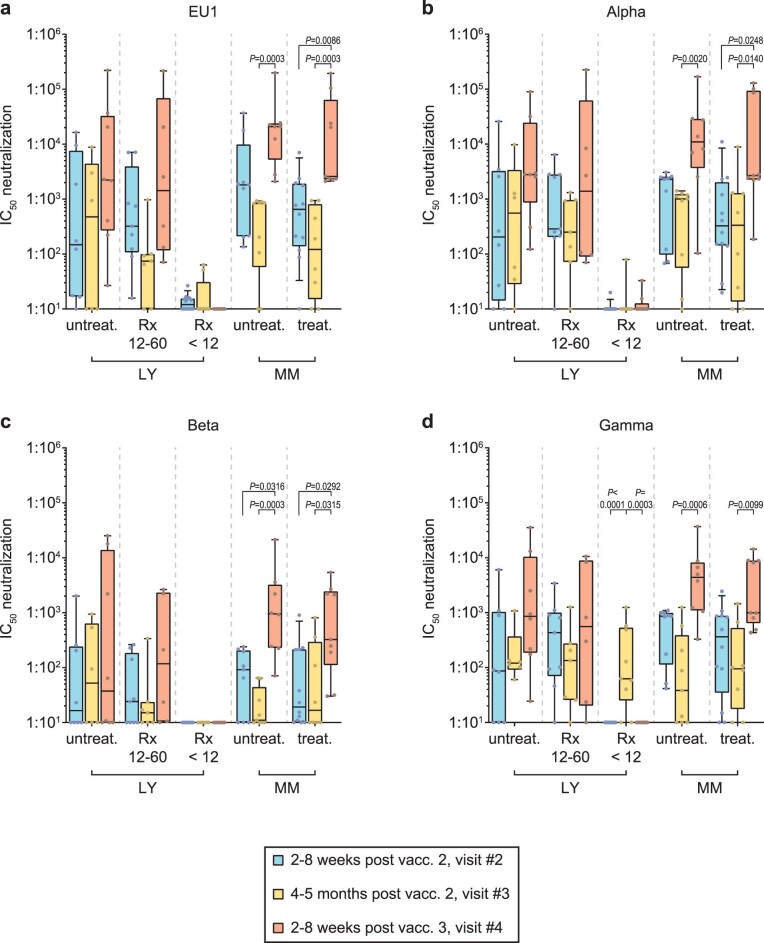
Longitudinal comparison of infection-neutralization activities against SARS-CoV-2 variants EU1, Alpha, Beta, and Gamma in subgroups of patients with hematologic neoplasia. Serum dilutions for half-maximal infection-neutralization capacities normalized to 10^7^ viral RNA copies (neutralization IC_50_-values) are depicted for different SARS-CoV-2 variants as box plots with median, bounds between upper and lower quartiles, and whiskers between the 10th and 90th percentiles. Differences between time points were tested for their statistical significance using the Kruskal-Wallis-test with Dunn’s multiple testing correction. Brackets show statistically significant differences. Absence of brackets or p-values indicates absence of significance. Sera from the following subgroups of patients were analyzed at visit #2 (blue), visit #3 (yellow) and visit #4 (red). **a**, Neutralization IC_50_-values for SARS-CoV-2 variant EU1. **b**, Neutralization IC_50_-values for VoC Alpha. **c**, Neutralization IC_50_-values for VoC Beta. **d**, Neutralization IC_50_-values for VoC Gamma: Untreated LY (visit #2/ visit #3/ visit #4: n = 8/6/8, respectively); Rx 12-60 (visit #2/ visit #3/ visit #4: n = 9/7/6, respectively); Rx <12 (visit #2/ visit #3/ visit #4: n = 14/9/9, respectively); untreated MM (visit #2/ visit #3/ visit #4: n = 9/9/8, respectively) and treated MM (visit #2/ visit #3/ visit #4: n = 12/8/9, respectively). Source data

As already observed in the longitudinal assessment of anti-SARS-CoV-2 spike IgG titers, analyses of neutralizing capacities also revealed considerable variability among the different subgroups of individuals with cancer according to disease and treatment administered. In untreated individuals with LY, little change in serum-neutralizing titers against all VoCs was noted from visit 2 to 4 (Fig. 3 and Extended Data Fig. 3). Individuals with MM, irrespective of their treatment status, showed either no change or slight to moderate reductions of serum-neutralizing capacity for SARS-CoV-2 variants from visit 2 to visit 3 (1.0- to 22.3-fold; Fig. 3 and Extended Data Fig. 3). In contrast to untreated individuals and Rx 12–60 individuals with LY, the neutralizing capacity against all variants in individuals with MM increased significantly after vaccination 3 (Fig. 3 and Extended Data Fig. 3), with individuals with untreated MM showing a level of neutralization capacity comparable to healthy individuals.

Taken together, individuals with malignant hematologic diseases who responded to COVID-19 mRNA vaccination with a humoral response displayed a serum neutralization activity that was, on average, only threefold lower than healthy individuals with time-, variant- and participant subgroup-specific patterns. Remarkably, a drastic increase of the neutralizing capacity per anti-spike IgG unit, which coincided with an enhanced antibody avidity, was observed early after the second COVID-19 vaccination in these vaccine-responsive individuals with hematologic malignancies compared to healthy individuals. Together, this underscores the importance of quantifying not only anti-spike IgG titers but also neutralization capacity and antibody avidity for a refined assessment of potential humoral correlates of clinical protection in these individuals with cancer.

### Vaccinated individuals mount SARS-CoV-2 spike-specific T cell responses

To examine the vaccine-induced SARS-CoV-2-specific T cell response, we applied an IFNγ ELISpot assay. To assess T cell responses before and after two-dose standard vaccination in individuals with cancer and matched healthy controls (that is, at visits 1 and 2), peripheral blood mononuclear cells (PBMCs) were stimulated with pools of overlapping peptides derived from the spike protein of the Delta VoC and a set of reference antigens in a total of 53 individuals with hematologic malignancies and 12 healthy individuals. Because some individuals with cancer, especially those with chronic lymphocytic leukemia, had high absolute and relative numbers of malignant lymphocytes in peripheral blood, we expressed the results as T cell-normalized spot-forming units (SFU) per million PBMCs.

**Fig. 4:**
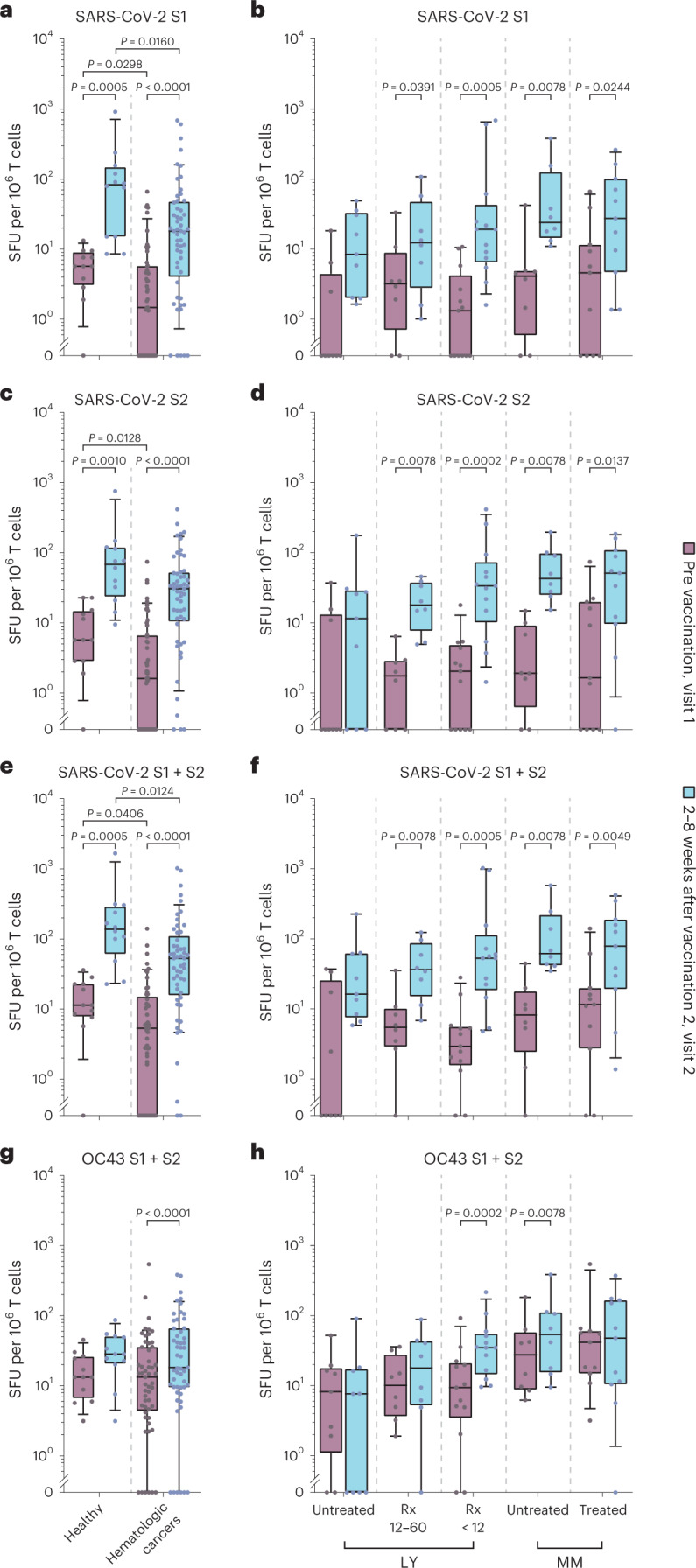
SARS-CoV-2- and OC43-specific T cell responses in individuals with hematologic malignancies before and after two-dose COVID-19 mRNA vaccination. T cell responses analyzed by IFNγ ELISpot and expressed as SFU per 10^6^ T cells are shown as box plots with median, bounds between upper and lower quartiles and whiskers between the 10th and 90th percentiles. Samples were obtained before vaccination 1 (visit 1) or at 2–8 weeks after vaccination 2 (visit 2). Differences between time points were analyzed for statistical significance using the Wilcoxon matched-pairs signed-rank test, and differences between healthy donors and individuals with hematologic malignancies were analyzed using the Mann–Whitney test. Brackets show statistically significant differences, and precise numerical *P* values are indicated. Absence of brackets or *P* values indicates absence of statistical significance. **a**,**b**, T cell responses to SARS-CoV-2 spike S1. **c**,**d**, T cell responses to SARS-CoV-2 spike S2. **e**,**f**, Sum of T cell responses to SARS-CoV-2 spike S1 and S2. **g**,**h**, Sum of T cell responses to human coronavirus OC43 spike S1 and S2. Results in **a**, **c**, **e** and **g** are shown for infection-naive healthy individuals (visit 1: *N* = 12/visit 2: *N* = 12) versus individuals with hematologic malignancies (visit 1: *N* = 53/visit 2: *N* = 53). Results in **b**, **d**, **f** and **h** are shown for infection-naive individuals with hematologic malignancies split into five subgroups: individuals with LY never treated with Rx or last treated more than 5 years before vaccination (untreated), individuals with LY last treated with Rx 12–60 months before vaccination (Rx 12–60), individuals with LY treated with Rx within 12 months before vaccination (LY < 12), individuals with MM not receiving therapy at the time of vaccination (untreated) and individuals with MM receiving therapy at the time of vaccination (treated). The number of individuals with hematologic neoplasia in these subgroups was as follows: untreated LY (visit 1, *N* = 9; visit 2, *N* = 9)/Rx 12–60 (visit 1, *N* = 8; visit 2, *N* = 8)/Rx < 12 (visit 1, *N* = 13; visit 2, *N* = 13)/untreated MM (visit 1, *N* = 8; visit 2, *N* = 8)/treated MM (visit 1, *N* = 11; visit 2, *N* = 11). Source data

**Extended Data Fig. 4:**
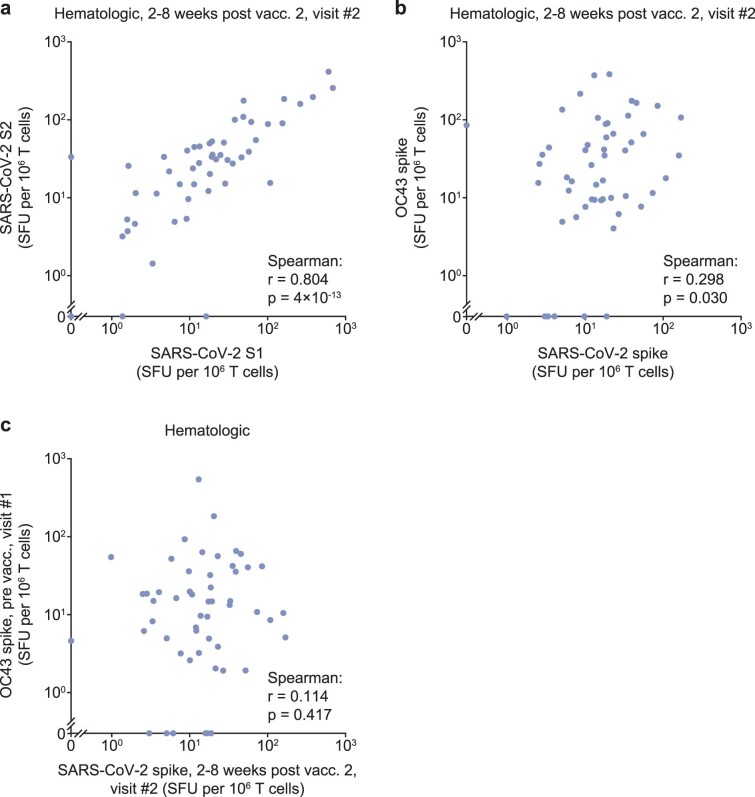
Correlation analysis of T-cell and antibody responses in hematologic patients at different time points before and after COVID-19 vaccination. Dot plots of T-cell responses analyzed by IFN-γ ELISpot and expressed as spot-forming units (SFU) per 10^6 ^T cells as well as anti-SARS-CoV-2 spike S1 domain antibody responses in BAU/mL. Two-tailed Spearman’s rank correlation analysis was performed to calculate correlation coefficients (r) and analyze statistical significance. **a**, Correlation of T-cell responses to peptide pools for SARS-CoV-2 VoC delta spike S1 and S2 domain in 53 patients with hematologic neoplasia at 2-8 weeks post vaccination #2. **b**, Correlation of T-cell responses to peptide pools for Delta spike or for human coronavirus (hCoV) OC43 in 53 patients with hematologic neoplasia at 2-8 weeks post vaccination #2. **c**, Correlation of T-cell responses to peptide pools for Delta spike, 2-8 weeks post vaccination #2, and for hCoV-OC43 prior to vaccination in 53 patients with different hematologic neoplasia. Source data

Delta spike-specific T cells increased in the majority of individuals with cancer and healthy individuals after vaccination 2 (Fig. 4a,c,e). At visit 2, 85% (45 of 53 participants) of all individuals with cancer had an increase in the frequency of Delta spike-specific IFNγ-secreting T cells compared to prevaccination levels. In 76% of individuals with cancer (40 of 53 participants), this increase amounted to 10 or more SFU per million T cells, and the median response increased from 5 to 50 SFU per million cells compared to the prevaccination baseline (Fig. 4e). The overall response was equally distributed to the S1 and S2 moieties of the Delta spike antigen (Fig. 4a,c), and these two responses were strongly correlated (Extended Data Fig. 4a). This suggests that the vaccination response was not predominantly shaped by individual limitations in T cell diversity. In comparison, 100% of healthy individuals (12 of 12) showed an enhanced frequency of Delta spike-specific IFNγ-secreting T cells at visit 2 compared to prevaccination visit 1, and their median responses to S1 and S2 spike peptides at visit 2 were 4.6- and 2.2-fold higher than those observed in individuals with cancer (Fig. 4a,c). Overall, this demonstrates a robust development of a cellular immune response after two mRNA vaccinations in most individuals with hematologic cancers.

**Extended Data Fig. 5:**
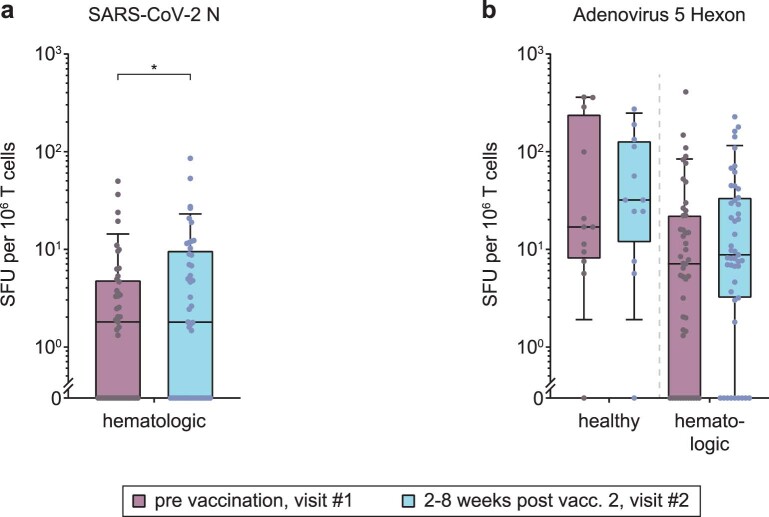
SARS-CoV-2 nucleocapsid- and adenovirus 5 hexon-specific T-cell responses in healthy individuals and patients with hematologic malignancies before and after the second COVID-19 vaccination. T-cell responses analyzed by IFN-γ ELISpot and expressed as spot-forming units (SFU) per 10^6 ^T cells are shown as box plots with median, bounds between upper and lower quartiles, and whiskers between the 10th and 90th percentiles. Results from infection-naive healthy individuals and patients with hematologic malignancies before vaccination (visit #1, purple) and after the second vaccine dose (visit #2, blue) are shown in (**a**, **b**): Responses to a peptide pool for SARS-CoV-2 nucleocapsid (hematological patients: visit #1/visit #2: n = 45/45, respectively) are shown in (**a**), and to a peptide pool for adenovirus 5 hexon protein (healthy individuals: visit #1/visit #2: n = 12/12, respectively; hematological patients: visit #1/visit #2: n = 47/47, respectively; the six patients who had received a dose of the adenoviral AstraZeneca vaccine were excluded) in (**b**). Brackets show statistically significant differences. Absence of brackets or p-values indicates absence of significance. Source data

We also analyzed the concomitant T cell response to the spike antigen of the endemic, seasonal β-coronavirus OC43. At visit 1, OC43 spike-specific T cells were detectable at slightly higher levels than SARS-CoV-2 spike-specific T cells (Fig. 4e,g), consistent with OC43 endemicity^51–53^. OC43-specific T cells were slightly increased at visit 2 in individuals with cancer and healthy individuals (1.4-fold and 2.1-fold higher median response, respectively; Fig. 4g), but this increase was expectedly less pronounced than the vaccine-induced increase in SARS-CoV-2 spike-specific T cells (Fig. 4e). Visit 2 responses to SARS-CoV-2 and OC43 spike were correlated only to a limited extent in individuals with cancer (Extended Data Fig. 4b). Moreover, the prevaccination response to OC43 in individuals with hematologic malignancies did not predict the response to SARS-CoV-2 after vaccination (Extended Data Fig. 4c). Thus, T cell cross-reactivity among these β-coronaviruses appears to be present but plays a minor role in shaping outcomes of COVID-19 vaccination. The response to SARS-CoV-2 nucleocapsid (Extended Data Fig. 5a) remained low before and after vaccination (median of 1.8 SFU at visits 1 and 2), indicating the absence of intermittent SARS-CoV-2 infection and corresponding to the observed negative results for the detection of anti-nucleocapsid IgG in all sera (Table 1). T cell responses to adenovirus hexon protein were detectable in most individuals with cancer and only slightly increased from visit 1 to visit 2 (Extended Data Fig. 5b). In contrast to some previous studies^17,20,23,54,55^, uncorrected background signals in our ELISpot approach were very low (0 spots in 83% of all individuals with cancer and in 91% in the subgroup of individuals with LY at visit 1), underscoring that bona fide antigen-specific T cells were detected in our analyses with high confidence.

**Extended Data Fig. 6:**
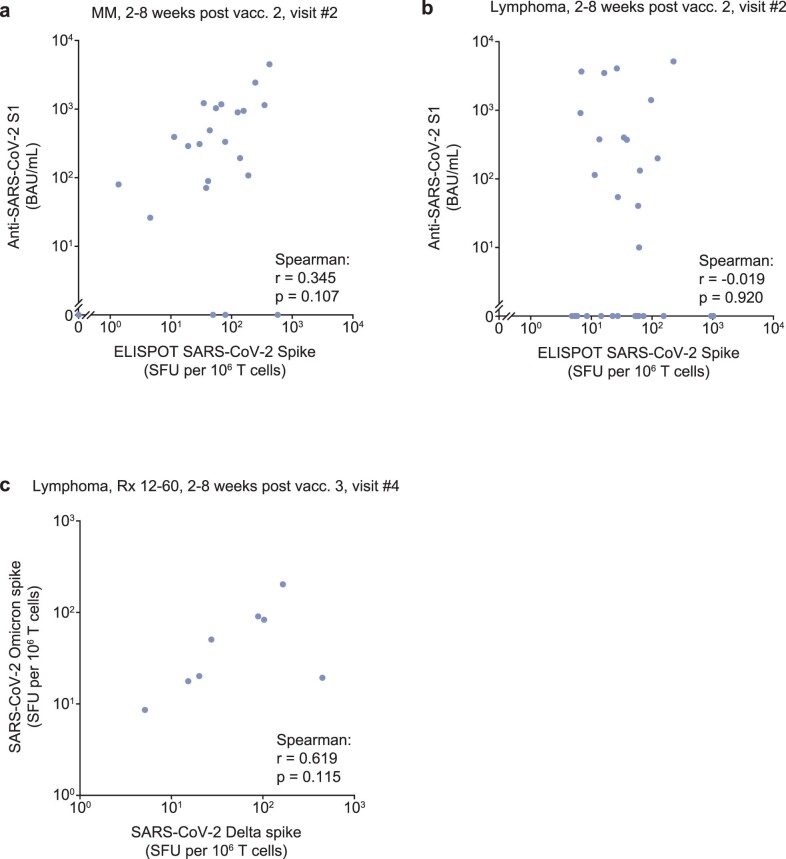
Correlation analysis of T-cell and antibody responses in patients with MM or LY at different time points after COVID-19 vaccination. Correlation of T-cell responses to peptide pools for Delta spike and levels of antibodies against the spike S1-domain 2-8 weeks post vaccination #2 in 19 patients with MM. **b**, Correlation of T-cell responses to peptide pools for Delta spike and levels of antibodies against the spike S1-domain 2-8 weeks post vaccination #2 in 30 patients with LY. **c**, Correlation of T-cell responses to peptide pools for spike from Delta and Omicron (BA.1) 2-8 weeks post vaccination #3 in 8 patients with LY last treated with Rx 12-60 months before vaccination. Source data

The T cell response was separately analyzed in 49 individuals with cancer in this cohort who could be allocated to one of the five subgroups with hematologic malignancies, as described above. An increase in the median T cell response was apparent in all five subgroups and was statistically significant in four of five subgroups for SARS-CoV-2 Delta S1, S2 and the total spike-specific response (Fig. 4b,d,f). An increase in OC43 spike-specific T cells was detected in two of five subgroups (Fig. 4h). The weakest response to the Delta spike protein was observed in untreated individuals with LY, but even in this group, an increase in total spike-specific T cells from a median of 0 SFU (range of 0–35) to 16 SFU (range of 6–224) was observed, with six of nine individuals with cancer showing increased spike-specific T cells (Fig. 4f). Of particular note, all 13 individuals with cancer from the Rx < 12 LY subgroup who were analyzed had detectable T cells against SARS-CoV-2 Delta spike protein (median of 53 SFU, range of 5–1,021; Fig. 4f), although none of these individuals had a vaccine-induced humoral immune response above background (Figs. 1c and 3). In the other four subgroups of individuals with cancer, a solid vaccination-induced T cell response was observed, representing an increase from a visit 1 subgroup-specific median baseline of 3–11 SFU to a visit 2 median response of 36–78 SFU (Fig. 4b,d,f). It is worth mentioning that the SARS-CoV-2 spike-specific T cell response after vaccination was very similar in individuals with MM receiving treatment (median of 78 SFU, range of 1–421) and in untreated individuals with MM (median of 62 SFU, range of 35–577). There was a trend of a positive correlation between spike-specific IgG and T cell responses in individuals with MM (Extended Data Fig. 6a) but not in individuals with LY (Extended Data Fig. 6b). The latter appeared to be able to mount a spike-specific T cell response even in the absence of B cells or a corresponding antibody response. Of note, after the booster dose at visit 4, T cell responses to Delta and Omicron (BA.1) spike peptides appeared closely correlated in seven of eight donors, with one individual scoring as an outlier who recognized Delta much better than Omicron (BA.1; Extended Data Fig. 6c).

**Extended Data Fig. 7:**
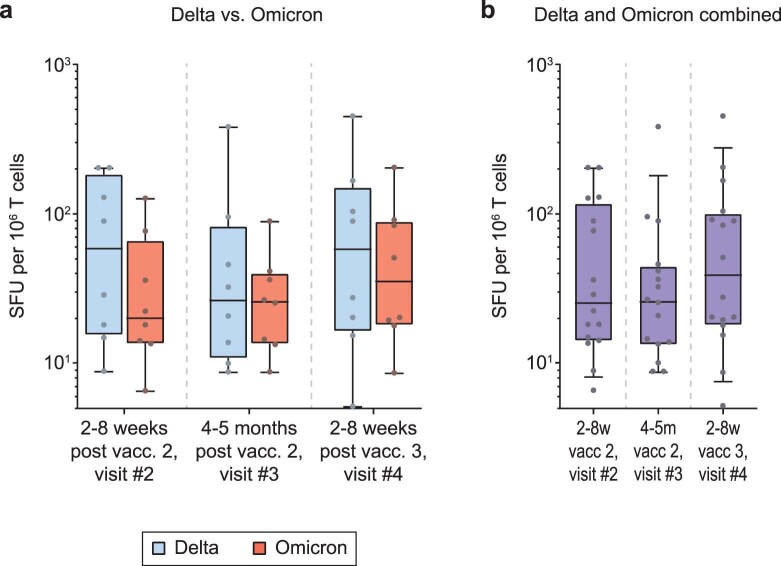
T-cell responses to VoCs Delta and Omicron in patients with lymphomas at different time points after the second and third COVID-19 mRNA vaccination. Responses to peptide pools spanning Delta spike (S1 + S2) or Omicron (BA.1) spike (S1 + S2) are shown in patients (n = 8) who received Rx treatment 12–60 months before vaccination are shown in (**a**) and combined responses to Delta and Omicron (BA.1) spike peptides in (**b**). Patients were studied 2-8 weeks post vaccination #2 (visit #2), 4-5 months post vaccination #2 (visit #3), and 2-8 weeks post vaccination #3 (visit #4). Differences between time points were analyzed for statistical significance using the Friedman test with Dunn’s multiple testing correction. However, no statistically significant differences were detected. Source data

To assess the sustainability of the T cell response and the effect of a third vaccination, we performed longitudinal (visits 2, 3 and 4) ELISpot analyses in eight individuals with LY who had received their last Rx treatment within 12–60 months before the first vaccination. For this analysis, T cells were tested against spike peptide pools from the VoCs Delta or Omicron (BA.1), because BA.1 had started to become the dominant VoC in Germany at the time of visit 4. Stimulation with the Delta peptide pools (S1 and S2) generally elicited a marginally higher T cell response than the Omicron (BA.1) peptide pools (S1 and S2) at visits 2 and 4, although these differences did not reach statistical significance (Extended Data Fig. 7a). When analyzing T cell responses to Delta and Omicron (BA.1) VoCs between visit 3 and visit 4 in a combined fashion, a positive trend of the booster vaccination was observed (Extended Data Fig. 7b).

In summary, COVID-19 vaccination of individuals with hematologic malignancies elicits potent T cell responses even in the absence of humoral responses in individuals recently treated with Rx. This response may contribute to the control of variants with pronounced humoral escape properties, including VoCs Omicron and Beta. However, in individual cases, the vaccine-induced T cell response might target epitopes in SARS-CoV-2 VoCs that are not conserved in Omicron (BA.1).

### Correlative analyses of vaccine responses and blood values

Next, we performed correlation analyses of vaccine-induced humoral and T cell responses to the total IgG concentrations and the counts of certain peripheral blood cell populations obtained from health records from individuals with hematologic malignancies.

**Fig. 5:**
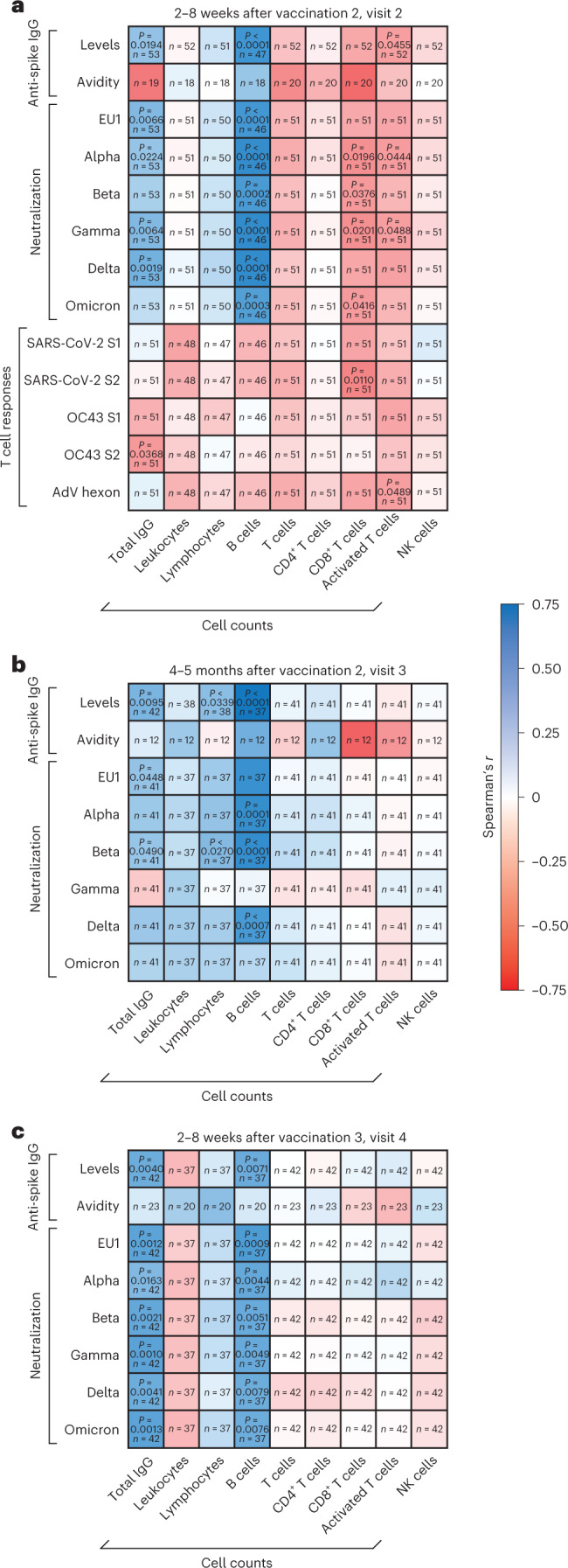
Comparison of humoral and T cell responses, leukocyte and leukocyte subgroup counts and total serum IgG in individuals with hematologic malignancies. **a**–**c**, IgG-type anti-SARS-CoV-2 spike levels, antibody avidity, serum neutralization activity against different SARS-CoV-2 variants and specific T cell responses against peptides derived from SARS-CoV-2 and OC43 spike and adenovirus (AdV) 5 hexon protein measured in individuals with hematologic malignancies were compared to counts of leukocytes, lymphocytes, B cells, T cells, CD4^+^ T cells, CD8^+^ T cells, activated T cells and natural killer (NK) cells and total serum IgG concentrations. Spearman’s correlation analysis was performed using an asymptotic two-sided test of the null hypothesis *r* = 0 versus *r* ≠ 0 based on the *t* distribution with *n* – 2 d.f. Spearman’s correlation coefficients (*r*) between SARS-CoV-2-specific immune responses and cell counts as well as total IgG concentrations are depicted as heat maps for the following time points: 2–8 weeks after vaccination 2 (visit 2; **a**), 4–5 months after vaccination 2 (visit 3; **b**) and 2–8 weeks after vaccination 3 (visit 4; **c**). *P* values are depicted for all significant correlations. Absence of *P* values indicates absence of significance; *n* indicates the number of pairs analyzed. Source data

At all time points after the second and third COVID-19 vaccinations, anti-SARS-CoV-2 levels and neutralization responses against the majority of SARS-CoV-2 variants correlated significantly with both B cell numbers and total IgG concentrations (Fig. 5a–c). At visit 4, the correlation of total IgG and infection-neutralizing titers was highly significant for all variants tested, including immune escape VoCs Omicron and Beta (Fig. 5c). Shortly after the second vaccination (visit 2), there was a trend toward a negative correlation between infection neutralization activities and CD8^+^ T cell counts that was statistically significant for VoCs Alpha, Beta, Gamma and Omicron (Fig. 5a). However, this negative correlation could not be observed at visits 3 (Fig. 5b) or 4 (Fig. 5c).

**Extended Data Fig. 8:**
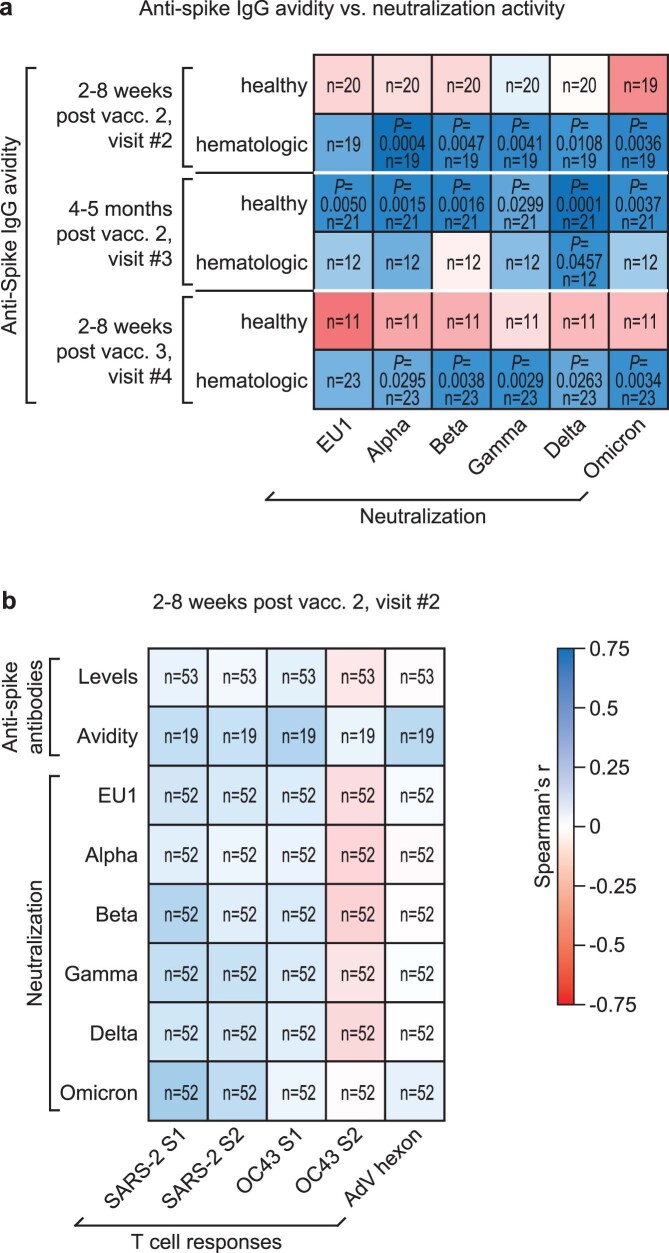
Comparison of vaccine-induced neutralization responses, anti-spike IgG antibody avidities and T-cell responses. **a**, IgG-type anti-spike antibody avidities were compared to serum neutralization activities against different SARS-CoV-2 variants in healthy individuals and hematologic patients 2-8 weeks after vaccination #2 (visit #2), 4-5 months after vaccination #2 (visit #3), and 2-8 weeks after vaccination #3 (visit #4). **b**, 2-9 weeks after vaccination #2 (visit #2) anti-SARS-CoV-2 spike antibody levels, antibody avidity and serum neutralization activity against different SARS-CoV-2 variants were compared with specific T-cell responses against peptides derived from SARS-CoV-2 and OC43 spike as well as adenovirus 5 hexon protein in hematologic patients. Spearman’s correlation analysis in (**a**, **b**) was performed using an asymptotic two-sided test of the null hypothesis r = 0 vs r ≠ 0 based on the t distribution with n-2 degrees of freedom. Spearman’s correlation coefficients (r) are depicted as heatmaps for the indicated time-points. P-values are depicted for all significant correlations. Absence of p-values indicates absence of significance. n – number of pairs analyzed. Source data

Shortly after the second and third vaccinations, antibody avidities were correlated with neutralizing activities in sera from individuals with hematologic malignancies but surprisingly not in samples from healthy individuals (Extended Data Fig. 8a). By contrast, antibody avidities measured in sera from healthy individuals correlated significantly with neutralization responses only at visit 3 (Extended Data Fig. 8a). Together with the observation that individuals with hematologic malignancies show higher neutralization potencies per anti-spike IgG unit shortly after the second vaccination than healthy individuals (Fig. 2d), these results indicate that the exposure to COVID-19 mRNA vaccines leads to a rather rapid development of highly efficient antibodies to spike in this group of individuals with cancer. Of note, anti-spike IgG levels, avidities and neutralization responses against SARS-CoV-2 in individuals with hematologic malignancies did not or only weakly correlate with T cell responses against SARS-CoV-2 spike, HCoV OC43 spike and adenovirus hexon protein at visit 2 (Extended Data Fig. 8b).

In conclusion, our analyses show a general positive correlation between B cell numbers and concentrations of antibodies to SARS-CoV-2 as well as neutralization responses in vaccinated individuals with hematologic malignancies. High CD8^+^ T cell numbers coincided with lower infection neutralization capacities but only shortly after the second vaccination. Furthermore, anti-spike IgG avidities correlated strongly with neutralization responses in individuals with hematologic malignancies shortly after the second and third vaccinations.

### Clinical presentation during breakthrough infections

Between 16 August 2021 and 25 July 2022, 13 vaccinated individuals in our hematologic cohort were diagnosed with a SARS-CoV-2 breakthrough infection. Seven of these individuals were LY Rx < 12 individuals, of which two had severe COVID-19 and were hospitalized, required oxygen (but no mechanical ventilation) and received either a combined treatment with casirivimab, imdevimab and tocilizumab or sotrovimab, two experienced moderate symptoms, and three experienced mild symptoms. Mild COVID-19 was also diagnosed in 2 untreated individuals with LY, 2 treated individuals with LY, and 2 untreated individuals with MM. Additionally, four individuals had a second breakthrough infection, one with moderate COVID-19 and three with mild COVID-19 (Extended Data Table 2). Based on prevalence data for VoCs during the summer and fall of 2021 and winter and spring 2022 in Germany, 4 of the breakthrough infections were likely caused by VoC Delta, and 13 were likely caused by VoC Omicron subvariants BA.1, BA.2, BA.4 and BA.5. In particular, the group of individuals with LY with Rx < 12 with virtually absent humoral vaccine responses was intriguing, because five of seven individuals did not require hospitalization for this infection event. No COVID-19-related death was seen in our group of individuals with hematologic malignancies.

## Discussion

Using a longitudinal approach, we studied the humoral and T cell immune responses elicited by two and three vaccinations (mostly with BNT162b2 mRNA) in a cohort of individuals with different B cell LYs and MM. Time-resolved, parallel assessment of titers of antibody to SARS-CoV-2 spike protein, antibody avidity and neutralization capacity to six authentic, replication-competent viruses, and SARS-CoV-2- and HCoV OC43-directed T cell responses allowed us to obtain a comprehensive picture of COVID-19 vaccine-induced immune responses in these individuals with hematologic cancers relative to a group of matched healthy individuals.

In the current study, we report six key findings. First, most individuals with a hematologic malignancy who are capable of developing a vaccine-induced antibody response mount an infection neutralization capacity against SARS-CoV-2 variants that is only slightly lower than in healthy individuals (reduced, on average, by 2.5-, 6.4- and 4.1-fold) despite markedly lower titers of antibody to the spike protein in the former group (reduced 49.1-, 23.6- and 9.2-fold at visits 2, 3 and 4, respectively). Second, in individuals with hematologic malignancies, the neutralizing potency per anti-spike antibody unit is drastically enhanced (20.8-fold on average) early after the second vaccination compared to healthy individuals. Third, the avidity of serum IgG binding to the SARS-CoV-2 spike protein in these individuals is higher before the third COVID-19 vaccination than in healthy individuals. Fourth, in a direct comparison of all VoCs, Omicron (BA.1) and, to a lesser extent, Beta and Delta displayed the most pronounced humoral immune escape, in line with results recently described for healthy individuals following either vaccination or infection^12^. Fifth, the majority of the participants in our study, including individuals with LY receiving Rx treatment, mounted a robust vaccine-induced T cell response to two recent VoCs. Sixth, clinical presentation during breakthrough infections is consistent with a partially protective effect of vaccination in immunocompromised individuals with hematologic malignancies with no report of a COVID-19-related death in our cohort.

Nevertheless, our observations indicate that individuals with LY without a humoral immune response due to recent Rx treatment have a higher risk for symptomatic breakthrough infections. This group of highly immunosuppressed individuals might benefit from passive immunization through a preexposure prophylaxis with neutralizing mAbs to the spike protein. This approach seems feasible, although many of the currently available mAbs have experienced a loss of antiviral efficacy for recent VoCs^43–45^.

Analyzing vaccine-induced antibody responses in at-risk individuals with hematologic malignancies is especially important because those individuals often display an imbalance between humoral and cellular immunity, which can be attributed to disease-related lineage defects, Rx treatment or BTK inhibition. Consequently, it is critical to define parameters that best reflect vaccine-induced immunity.

Our multiparametric longitudinal analysis of vaccine-induced humoral immunity revealed that the quality of antibodies to the spike protein after the second vaccination was remarkably high in individuals with hematologic malignancies who developed antibodies of superior neutralizing quality, as reflected by the ratio of neutralizing titer per antibody unit. By contrast, healthy individuals required a third vaccination to reach a comparable neutralizing capacity. Consistently, increasing antibody avidities correlated strongly with neutralization responses shortly after the second vaccination in individuals with hematologic malignancies. A sudden increase in antibody avidity was noted in healthy individuals only following the third vaccination compared to a steady enhancement of avidity between visits 2 and 4 in our cancer cohort. While sampling time points do not perfectly match between these two non-contemporaneous cohorts, we demonstrate that even 68 additional days, on average, after the second vaccination in healthy individuals did not result in the same high avidity as in individuals with hematologic malignancies shortly after the second vaccination. In line with this finding, the neutralizing potency per anti-spike IgG unit was also significantly higher in individuals with hematologic malignancies at 2–8 weeks after the second vaccination than at 4–5 months and even 7 months in healthy individuals. Collectively, this highlights that differences in anti-spike IgG avidity and neutralizing capacity observed between the two cohorts cannot be accounted for by small differences in sampling time points.

We speculate that in vaccinated individuals with LY and MM, following only two exposures to the spike protein, the affinity maturation of memory B cells may be accelerated or the breadth of antibodies may be expanded by yet undefined mechanisms. In hematologic malignancies, a deregulation and constitutive expression or activation of proteins of the apolipoprotein B mRNA-editing enzyme catalytic polypeptide (APOBEC) family of deaminases, which are involved in oncogenesis, has been reported^56–61^. Canonical functions of these types of enzymes, particularly activation-induced cytidine deaminase (AID), are key to somatic hypermutation in B cells and thus the generation of high-affinity antibodies. We propose that higher activities of AID/APOBEC family members in B cells of these types of hematologic cancer could promote antibody maturation.

While the third vaccination was of importance in both groups to elevate antibody titers, we propose that the quality rather than the mere quantity of vaccine-induced spike-targeting antibodies is of exceptional importance and has a specific signature in individuals with hematologic malignancies. Anti-spike titers alone may thus underestimate the potency of the humoral response in COVID-19 vaccinees with LY or MM, and neutralization and antibody avidity provide additional depth to defining potential functional correlates of protection from severe COVID-19. Consistently, recent studies indicate that neutralizing antibody levels may be highly predictive of immune protection from symptomatic COVID-19 (refs. 11,62).

mRNA-based COVID-19 vaccines are known to induce spike-specific T cell responses in the majority of healthy recipients^36,63,64^. In individuals with cancer, more heterogeneous results have been reported after two vaccinations^15,17,20,23,24^. Our findings on vaccine-induced T cell responses in 85% of individuals with hematologic cancers are in line with a large British cancer cohort study^54^ that showed detectable responses in up to 77% of individuals and a cohort of individuals with hematologic cancers with response rates of 80% (refs. 20). Liebers et al. found spike-specific T cells in only 58% of individuals with B cell malignancies with Rx treatment^24^. These authors used low numbers of input cells in their ELISpot analysis, which may have led to an underestimation of functional spike-specific T cells^24^. Our finding of functional T cell responses in 12 of 13 individuals with hematologic cancer (92%) currently or recently treated with Rx supports the notion that two-dose COVID-19 mRNA vaccination may indeed be of high benefit even for individuals who are unable to develop antibody responses. Our limited clinical observations during breakthrough infections indicate that even individuals with LY who are B cell deficient were largely protected from severe COVID-19, likely due to T cell-mediated immunity.

The majority of our two-dose-vaccinated individuals with MM (89%, 16 of 18) developed a detectable SARS-CoV-2 spike-specific T cell response, while Enßle et al. reported considerably lower numbers (34%)^23^. Of note, only 71% of their vaccinated healthy individuals (controls) scored positive, which stands in contrast to findings by us and others^63–65^. Possible explanations for these discrepancies include a high background signal in their IFNγ ELISpot assay and their use of incomplete peptide pools^23^. Our results suggest that also the majority of vaccinated individuals with MM raises both efficient spike-specific T cell responses and neutralizing antibody responses.

The strengths of our study include the longitudinal sampling of the clinically well-characterized cohort of individuals with cancer and the matched group of healthcare workers and the expanded methodological approach, including live virus neutralization assays against multiple VoCs and the quantification of anti-spike IgG avidity. Limitations of our study include the fairly small number of participating individuals with hematologic cancer (*N* = 60). The study lacks a contemporaneous control set with other hematologic malignancies or solid tumors. For correlation analyses, adjustments for multiple testing were not performed, making it difficult to exclude potential confounders.

Based on our current results, the multiparametric assessment of quantity and quality of COVID-19 immunity elicited by vaccines and infection should be the future standard in larger longitudinal studies seeking to strengthen the role of individual quantitative markers as correlates of protection in individuals with hematologic malignancies in light of newly emerging VoCs of this pandemic virus.

## Methods

### Participants and samples

We conducted a monocentric, observational cohort study that commenced recruitment in March 2021 and continued to enroll participants until May 2021. We report results generated during extensive analyses of participant samples received within this study. Because the course of the pandemic and the sudden availability of COVID-19 vaccines for individuals with cancer was not predictable initially, the consent of our participants for an additional draw of blood during a regular outpatient visit was obtained through signing the informed consent form of the Biobank (FREEZE) of the Medical Center of the Freiburg University. Subsequently, all individuals signed the informed consent form of this study. Participants who signed the Biobank (FREEZE) consent before the study-specific informed consent were considered ‘retrospective’ in this specific case. The timing of blood draws in relation to vaccination date was ‘prospective’ in all cases. The study was initiated at the Biobank (FREEZE) on 8 March 2021; the local ethics committee received all documents on 1 June 2021.

The first inclusion date of a study participant was 9 March 2021, and the last inclusion date was 17 May 2021.

The following were the study inclusion criteria: Participants must not have received any prior COVID-19 vaccination and must have one of the following diagnoses (and therapy): B cell Non-Hodgkin’s lymphoma (follicular lymphoma, mantle cell lymphoma, marginal zone lymphoma, diffuse large B cell lymphoma and chronic lymphatic leukemia) without treatment (‘watch and wait’), B cell Non-Hodgkin’s lymphoma less than 12 months after therapy with mAbs to CD20 (Rx, obinutuzumab), B cell Non-Hodgkin’s lymphoma 12–60 months after therapy with mAbs to CD20, chronic lymphathic leukemia with BTK or BCL-2 inhibition (venetoclax, ibrutinib), MM (watch and wait, treatment with lenalidomide and bortezomib or treatment with daratumumab, ixazomib and carfilzomib) or non-small cell lung cancer under PD1/PD-L1 checkpoint inhibition (durvalumab, pembrolizumab and nivolumab).

All individuals fulfilling these inclusion criteria and who visited our outpatient clinics between March and May 2021 were given the chance to participate.

The primary endpoint was defined as evaluation of the immunogenicity of COVID-19 vaccination 2–8 weeks after the second vaccination, as confirmed by the presence of spike-specific antibodies to SARS-CoV-2 (Fig.1b). The secondary endpoints focused on a profound analysis of the vaccine-induced immune response. When we started our sample collection, no prior published data on COVID-19 vaccine responses in individuals with cancer were available, and, therefore, no precise sample size could be calculated. Having 80 participants as the goal for recruitment was a best guess estimate from how many individuals with the respective diagnosis were seen in our outpatient clinics from March to May 2021. Because our study design required a blood draw before the first COVID-19 vaccination and the scheduling of vaccination was not part of this study (participants were vaccinated by their primary care physician or national vaccination centers), no further participants could be included in the study after May 2021, resulting in the inclusion of 60 individuals with hematologic malignancies in total. The few recruited individuals with non-small cell lung cancer were disregarded in this report focusing on hematologic malignancies. Addition of further time points for blood draws as well as further analyses with emerging VoCs were in agreement with the local ethics committee.

This study was conducted in accordance with the ethical principles of the Declaration of Helsinki, Good Clinical Practice and applicable regulatory requirements. Adult participants provided written consent to participate in this study or to contribute samples to the Biobank, including agreement to the deposition of pseudonymized data. Participants were not financially compensated. Due to the observational design of this study, no randomization was performed.

This study was approved by the local Ethics Committee (21-1386) and registered at the Paul-Ehrlich Institute (NIS599) and Deutsches Register Klinischer Studien (DRKS00025901). Clinical information regarding participant medical histories was obtained from the hospital digital medical file system, allowing for accurate follow-up. Cancer type was defined according to the International Classification of Disease (10th revision) diagnostic codes. The registered data were abstracted from medical files by board-approved hematologists/oncologists. Details of time points of vaccination and blood sample collection are depicted in Fig. 1a and Table 1. Prevaccination samples were collected at a median of 2 d before vaccination 1 (interquartile range (IQR): 1–6; visit 1). Subsequent samples were collected 35 d (IQR: 41–30; 2–8 weeks) after vaccination 2 (visit 2), 151 d (IQR: 164–137; 4–5 months) after vaccination 2 (visit 3) and 41 d (IQR: 56–31; 2–8 weeks) after vaccination 3 (visit 4). Vaccinations 1 and 2 were administered 42 d (IQR: 42–24) apart, and vaccinations 2 and 3 were administered 189 d (IQR: 207–174) apart. The last blood sample included in this report was drawn on 20 January 2022.

For the non-contemporaneous reference cohort of healthy individuals^12^, blood samples were collected either 14 d (IQR: 13–14; 2–8 weeks), 103 d (IQR: 98–105; 4–5 months) or 210 d (IQR: 208.5–217.5; 7 months) after vaccination 2 or 14 d (IQR: 13–15.25; 2–8 weeks) after vaccination 3.

Baseline data included age, sex, ethnicity, cancer and treatment type at time of first dose of vaccination, dates of vaccinations, type of vaccine and interval of the last treatment in relation to the first vaccine dose. Autologous stem cell transplantation at any time before vaccination was also registered (Table 1).

We considered individuals to have a breakthrough SARS-CoV-2 infection if they tested positive for SARS-CoV-2 RNA by real-time PCR in a respiratory swab.

We collected 36 ml of EDTA blood and 7.5 ml of serum from 60 individuals with hematologic neoplasia (for detailed participant characteristics, see Table 1). Plasma was derived from peripheral blood samples by centrifugation and frozen immediately at −80 °C. PBMCs were isolated using a lymphocyte separation density gradient and immediately frozen in liquid nitrogen.

Data for antibody responses and PBMCs for T cell assays from vaccinated, infection-naive healthy individuals were obtained from a previously described cohort^12^. This cohort included healthcare workers vaccinated with BNT162b2 mRNA. This study was approved by the local ethics committee (ethics vote 476/20 and 26/21S-SR), and participants provided written informed consent to study participation and biobanking. To account for the heterogeneity of both cohorts, we selected cases from the latter group based on age and time intervals to vaccinations and sample collections.

### Quantitative antibody detection assays

IgG-type antibody responses to the S1 domain of SARS-CoV-2 spike antigen were quantified using the commercial, automized SARS-CoV-2 IgG II quant assay (Abbott). Levels of IgG-type antibodies to SARS-CoV-2 spike S1 domain in healthy individuals were previously measured using the anti-SARS-CoV-2 QuantiVac enzyme-linked immunosorbent assay (ELISA; IgG; EuroImmun)^12^. To investigate possible differences in the quantitative detection of antibody levels between the two assays, we compared the BAU per ml in serum samples of 20 individuals with COVID-19 measured with both assays. Pearson correlation analysis showed strong (*r* = 0.85) and significant (*P* ≤ 0.0001) correlation, and linear regression analysis revealed a conversion factor of 0.71 for quantitative results in BAU per ml between the two assays. This conversion factor was used to normalize and compare data for quantitative antibody measurements shown in Fig. 1b,c and Extended Data Fig. 1b,c.

### Antibody avidity assay

Binding strength of the SARS-Cov-2 IgG antibodies to the spike antigen of SARS-CoV-2 strain Wuhan-hu-1 were quantified by adaptation of the commercial IgG agile SARS-CoV-2 ELISA (Virion/Serion) using ammonium thiocyanate (Roth) as a chaotropic agent as described previously^66^. The following formula was used to calculate relative avidity: percent avidity = (IgG concentration, ammonium thiocyanate treated)/(IgG concentration, PBS treated) × 100.

### SARS-CoV-2 neutralization assay

High-titer virus stocks were generated and characterized as reported recently^12,67^. The following stocks of clinical isolates of different SARS-CoV-2 variants were used: GISAID EPI ISL 2450298 (EU1/B.1.177), 2095258 (VoC Alpha/B.1.1.7), 1752394 (VoC Beta/B.1.351), 2095178 (VoC Gamma/P.1/B.1.1.28.1), 2772700 (VoC Delta/B.1.617.2) and 7808190 (VoC Omicron (BA.1)/B.1.1.529). Infection neutralization activities in serum samples were quantified as described previously^12,47^.

### IFNγ ELISpot assays

IFNγ ELISpot assays were used to determine the frequency of virus peptide-specific T cells^68^. ELISPOT 96-well plates (MultiScreen MSIPN4510, Millipore) were pretreated with 35% ethanol in water, washed and coated overnight with anti-IFNγ according to the manufacturer’s protocol (antibody 1-D1K, Mabtech). The next day, cryopreserved PBMC samples were thawed and used in the assay within 2 h of thawing. PBMCs (250,000 cells per well) were co-incubated with pools of 15-mer peptides at 0.5 μg ml^–1^ per peptide (PepMix, JPT) in 200 μl of medium (RPMI 1640 (Gibco) with 5% human male AB serum (PAN Biotech)). Reactions were generally set up in triplicate wells; duplicate reactions were used when cell numbers were insufficient. Plates were developed by incubation with anti-IFNγ–biotin and streptavidin-alkaline phosphatase according to the manufacturer’s instructions (Mabtech). Spots were developed using the AP Conjugate Substrate kit (Bio-Rad). Spots were counted in an automated ELISpot reader (C.T.L. ImmunoSpot).

Peptide pools used in the ELISpot assays were protein-covering pools of 15-mer peptides overlapping in 11 amino acids: SARS-CoV-2 Delta variant spike protein parts 1 and 2 (first and second half of the amino acid sequence), SARS-CoV-2 (Wuhan-Hu-1) nucleoprotein, hCoV OC43 spike protein parts 1 and 2 and human adenovirus 5 hexon protein (all from JPT).

T cell responses expressed as SFU were normalized to the frequency of T cells in the individual PMBC samples. Normalization was mandatory because PBMCs collected from the different groups of individuals with hematologic cancers showed heterogeneous T cell abundance due to the high B cell lymphocytosis characteristic of certain lymphomas (for example, chronic lymphocytic leukemia).

### Statistics and reproducibility

Data and statistical analyses were performed in Prism 9 (GraphPad Software). Doses of virus required to infect 90% of cells and IC_50_ values for neutralization were calculated after normalized, sigmoidal dose–response curve approximation of the respective data. Precise numerical *P* values for all statistical analyses can be found in the respective figures and the Supplementary Information.

Data distribution was assumed to be not normal, but this was not formally tested.

No statistical method was used to predetermine sample size. When we started our participant recruitment and sample collection, no published data on COVID-19 vaccine responses in individuals with hematologic cancer were available.

Data from individuals positive for antibodies to SARS-CoV-2 nucleocapsid were excluded. The investigators were blinded to allocation during experiments and outcome assessment.

### Reporting summary

Further information on research design is available in the Nature Portfolio Reporting Summary linked to this article.

### Supplementary information

Supplementary Information

Reporting Summary

### Source data

Source Data Fig. 1 Statistical source data.

Source Data Fig. 2 Statistical source data.

Source Data Fig. 3 Statistical source data.

Source Data Fig. 4 Statistical source data.

Source Data Fig. 5 Statistical source data.

Source Data Extended Data Fig. 1 Statistical source data.

Source Data Extended Data Fig. 2 Statistical source data.

Source Data Extended Data Fig. 3 Statistical source data.

Source Data Extended Data Fig. 4 Statistical source data.

Source Data Extended Data Fig. 5 Statistical source data.

Source Data Extended Data Fig. 6 Statistical source data.

Source Data Extended Data Fig. 7 Statistical source data.

Source Data Extended Data Fig. 8 Statistical source data.

## Figures and Tables

**Extended Data Table 1:**
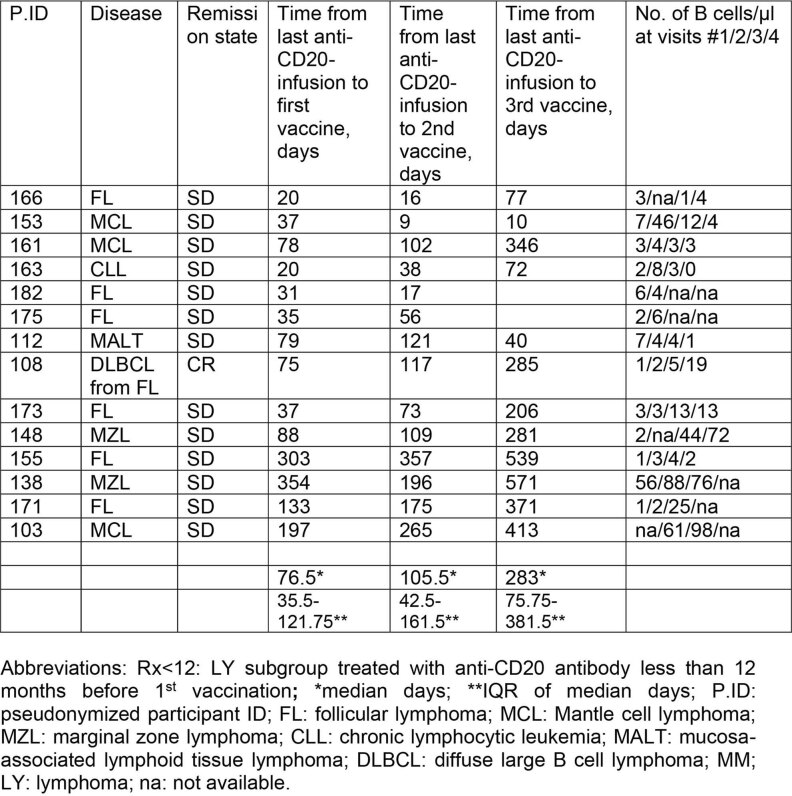
Remission state and time between last infusion of anti-CD20 antibody and COVID-19 vaccination in the Lymphoma (LY) subgroup Rx<12

Pseudonymized participant ID, disease, remission state, time from last anti-CD20 mAb infusion relative to the time-point of 1st, 2nd and 3rd vaccination, respectively, in days. Number of B cells per µl at visits #1-4 of the individual patient of this subgroup of 14 patients. “Remission state” is defined as the status of remission at the time of the first vaccination. Visits #1-4 are the time-points of blood sample collection as depicted in the time chart of the study in Fig. 1a. Visit #1: Pre-vaccination samples were collected shortly before vaccination #1. Visit #2: 2-8 weeks (median 35 days) after vaccination #2. Visit #3: 4-5 months (median 149 days) after vaccination #2. Visit #4: 2-8 weeks (median 40 days) after vaccination #3. Abbreviations: Rx<12: LY subgroup treated with anti-CD20 mAb less than 12 months before 1^st^ vaccination; *:median days; **:IQR of median days; P.ID: pseudonymized participant ID; LY: lymphoma; FL: follicular lymphoma; MCL: Mantle cell lymphoma; MZL: marginal zone lymphoma; CLL: chronic lymphocytic leukemia; MALT: mucosa-associated lymphoid tissue lymphoma; DLBCL: diffuse large B cell lymphoma; (Rx < 12): treated with rituximab <12 months before the 1^st^ vaccination.

**Extended Data Table 2:**
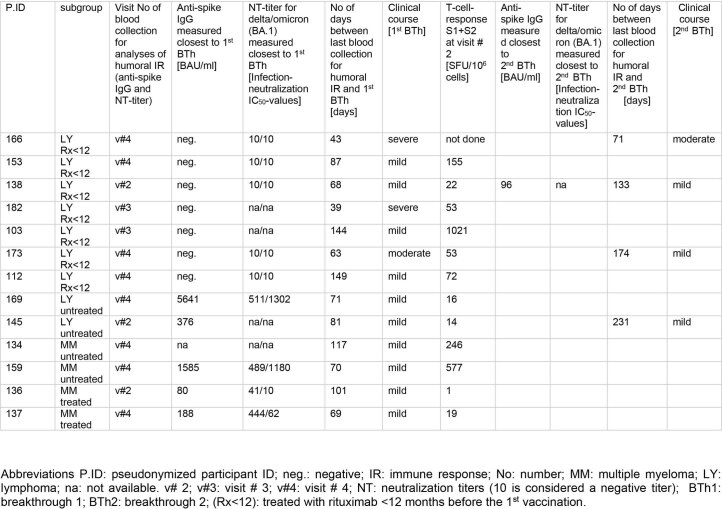
Anti-spike-IgG-levels, NT-titers for delta/omicron (BA.1) and T-cell response before breakthroughs

Pseudonymized participant ID, subgroup, visit number at which blood was taken for analyses of humoral immune response; anti-spike IgG, neutralization titers against Delta and Omicron (BA.1); number of days between last blood collection for analyses of humoral IR and breakthrough #1; clinical course of breakthrough #1; T-cell response at visit # 2; anti-spike IgG closest to breakthrough #2 if different from titer to breakthrough #1; neutralization titers against Delta and Omicron (BA.1) closest to breakthrough #2 if different from titer to breakthrough #1; number of days between last blood collection for analyses of humoral IR and breakthrough # 2 as well as the clinical course. Below the cutoff of 10 BAU/mL anti-spike-IgG levels are considered negative. Altogether, up to July 25, 2022 we documented 17 breakthroughs in 13 patients with four patients having each two breakthroughs. In the LY subgroup Rx<12 consisting of 14 patients altogether, we observed breakthroughs in seven patients (7/14 patients corresponding to 50%) of which three had two breakthroughs. None of the breakthroughs were lethal, most of them were of mild course. Abbreviations P.ID: pseudonymized participant ID; neg.: negative; IR: immune response; No: number; MM: multiple myeloma; LY: lymphoma; na: not available. v# 2; v#3: visit # 3; v#4: visit # 4; **NT**: neutralization titers (10 is considered a negative titer); **BTh1**: breakthrough #1; **BTh2**: breakthrough #2; (Rx < 12): treated with rituximab <12 months before the 1^st^ vaccination.

## Data Availability

Pseudonymized participant data, including participant record data and all primary data from measurements conducted, are available in a public repository (https://data.mendeley.com/datasets/z6dw96y8sw/1). The SARS-CoV-2 sequences and protein data are available under accession codes 6VXX (Protein Data Bank), MW717675.1 and MZ945494 (GenBank) and EPI_ISL_412971, EPI_ISL_2557176 and EPI_ISL_8768822.2 (GISAID). Source data are provided with this paper. All other data supporting the findings of this study are available from the corresponding author on reasonable request.
