# Supplementary material for: Potent high-avidity neutralizing antibodies and T cell responses after COVID-19 vaccination in individuals with B cell lymphoma and multiple myeloma
Source: Nat Cancer. 2022 Dec 21;4(1):81–95. doi: 10.1038/s43018-022-00502-x (PMC9886553; doi:10.1038/s43018-022-00502-x)
Supplement: Supplementary file 1 — Supplementary Information [file 43018_2022_502_MOESM1_ESM.pdf]

nature cancer

Article https://doi.org/10.1038/s43018-022-00502-x

Potent high-avidity neutralizing antibodies and T cell responses after COVID-19 vaccination in individuals with B cell lymphoma and multiple myeloma

In the format provided by the authors and unedited

STUDY PROTOCOL SYNOPSIS

**Study title**	Humoral and Cellular Immune Response of Commercially Available Vaccines Against SARS-CoV-2 in Patients with Hematologic and Solid Malignancies
**Protocol code**	
**EudraCT Number**	Not necessary
**Study design**	This study is a monocentric, observational retrospective and prospective cohort study in patients with malignant pathologies treated at the University Medical Center of Freiburg. The clinical study is planned and executed in adherence to rules for data evaluation.
**Study Type**	Monocentric, observational, retrospective and prospective data evaluation.
**Medical Condition**	Patients with certain Hematologic Malignancies and Patients with non-small cell lung cancer.
**Sponsor**	University of Freiburg Medical Center<br>Division of Hematology, Oncology & Stemcell transplantation
**Coordinating investigator**	Dr. med. Andrea Hafkemeyer<br>University of Freiburg Medical Center<br>Division of Hematology, Oncology & Stemcell transplantation<br>Hugstetter Straße 55<br>D - 79106 Freiburg<br>Germany
**Subinvestigators**	Dr. med. Christine Greil<br>Dr. med. univ. Khalid Shoumariyeh<br>Prof. Dr. med. Monika Engelhardt<br>Prof. Dr. med. Jürgen Finke<br>PD Dr. Reinhard Marks
**Hypotheses**	Our main hypothesis is that immune responses to COVID-19 vaccination differ among patients with certain hematologic and certain oncologic malignancies due to the disease itself and / or due to the appropriate therapy they receive (e.g. anti-CD20 monoclonal antibodies, Bruton´s tyrosine kinase- or BCL-2-inhibition, PD-1/PD-L1 checkpoint inhibition, immunomodulatory drugs).
**Rationale**	Vaccination may provide a sustainable termination of the current COVID-19 pandemic. Vaccines against SARS-CoV-2 have proven highly efficacious in randomized trials in healthy volunteers. Yet, the immunological correlates of protection are unknown and it is unclear if immunogenicity and protection is

	developed and preserved in patients with certain hematologic and certain oncologic malignancies with underlying immunodeficiency or immunosuppression due to therapy.
**Sample Size and Statistical analysis**	We expect to include approximately 80 participants all together in 5 subcohorts in this study.<br>Regarding the primary analyses, we assume that these sample sizes yield enough power for the intended comparisons.<br>Regarding the primary endpoint, immunogenicity of the COVID-19 vaccination (2)-8 weeks after the second vaccination as confirmed by the presence of anti-SARS-CoV-2-specific antibodies, counts and percentages with 95% confidence intervals (CI) per study subpopulation will be reported.
**Primary endpoint**	Immunogenicity of COVID-19 vaccination (2)-8 weeks after second vaccination as confirmed by the presence of anti-SARS-CoV-2-spike-specific antibodies (+ anti-NC-antibodies to assess previous COVID-19).
**Secondary endpoints**	• Immunogenicity of COVID-19 vaccination (2)-8 weeks after second vaccination as confirmed by the presence of SARS-CoV-2 -specific CD4+ T cells.<br>• Immunogenicity of COVID-19 vaccination (2)-8 weeks after second vaccination as confirmed by the presence of neutralizing antibodies against SARS-CoV-2 wildtype (D614G).<br>• Immunogenicity of the COVID-19 vaccination (2)-8 weeks after second vaccination as confirmed by the presence of neutralizing antibodies against different VOC PANGO lineages of SARS-CoV-2 (e.g., B.1.1.7, B1.351, P.1, B.1.617).<br>• Immunogenicity of COVID-19 vaccination 6, 12 and 18 months (+/-2 weeks) after first vaccination as confirmed by the presence of anti-SARS-CoV-2-specific antibodies.<br>• Differences in humoral and cellular immunogenicity in our patients with malignant pathologies within the different hematologic malignancies and between the treatments.
**Inclusion criteria**	Patients must not have received any prior COVID-19 vaccination and have one of the following diagnosis (and therapy):<br>• B-cell Non-Hodgkin's lymphoma (Follicular lymphoma, Mantle cell lymphoma, Marginal zone lymphoma, Diffuse large B-cell lymphoma, Chronic lymphatic leukemia) without treatment ("watch-and-wait"):<br>• B-cell Non-Hodgkin's lymphoma less than 6 months after therapy with anti-CD20 monoclonal antibodies (Rituximab, Obinutuzumab)<br>• B-cell Non-Hodgkin's lymphoma 6 - 60 months after therapy with anti-CD20 monoclonal antibodies<br>• Chronic lymphathic leukemia with Bruton´s tyrosine kinase- or BCL-

	2-inhibition (Venetoclax, Ibrutinib)<br>• Multiple Myeloma ("watch & wait"; treatment with Lenalidomide, Bortezomib; Daratumumab, Ixazomib, Carfilzomib)<br>• Non Small Cell Lung Cancer (NSCLC) under PD1/PD-L1-checkpoint inhibition (Durvalumab, Pembrolizumab, Nivolumab)
**Exclusion criteria**	An individual who meets any of the following criteria will be excluded from participation in this study:<br>• Any contraindications for the SARS-CoV-2 vaccination (according to the reccommendations of the "Ständige Impfkomission des Robert Koch Instituts").<br>• Refusal to participate.<br>• Patients unwilling to consent to saving and propagation of pseudonymized medical data for study reasons.<br>• Persons who can not give informed consent
**Investigational medicinal product**	All COVID-19 vaccines approved and used in Germany (Vaxzevria, AstraZeneca; COVID-19 Vaccine, Janssen; Comirnaty, BioNTech/Pfizer, COVID-19-Vaccine, Moderna).
**Study intervention**	Blood sampling (maximum of 50 ml per visit) at each of the 2 to max. 5 visits (study visits are planned at the time point of routine diagnostic appointments).<br>Note: The study has no influence on the vaccination process and procedure.
**Safety and Discontinuation Criteria**	Premature termination of individual participation:<br>• Withdrawal of informed consent<br>• Any other circumstance that makes the investigator believe that in the patient's own interest he/she should no longer participate in the study<br>• Significant violation of the study protocol<br>• Loss of contact, relocation, change of treating physician<br>• Subsequent occurrence of exclusion criteria (after enrollment)<br><br>Premature termination of the clinical trial if<br>• authorization is withdrawn by the competent authority or the favorable opinion is withdrawn by the ethics committee.<br>• occurrence of unexpected, significant, or unacceptable risk to subjects safety (benefit-risk-ratio is no longer favorable).
**Risk-benefit analysis**	**Risks of intervention**<br>Vaccination: The respective risks of the administered vaccine apply (see attached "Product Characteristics" of the respective vaccines). However, the administration of the vaccine and the risks that may occur are independent of the participation in the study.

	Blood sampling: low risk of thrombophlebitis or malposition when taking blood.<br><br>**Risks to privacy**<br>Subjects will be asked to provide personal health information. All attempts will be made to keep this information confidential according to regulatory requirements and national law. However, there is a chance that unauthorized persons will see the subjects personal health data.<br><br>**Risk mitigation**<br>Blood sampling will be performed only by an investigator or under his or her direction and supervision by medical personnel.<br>All study records will be kept in a locked file cabinet or maintained in a locked room at the participating clinical site. Electronic files will be and are already password protected. Only people who are involved in the conduct, oversight and monitoring of this trial will be allowed to access the personal health information that is collected.<br>Any publications from this trial will not use information that will identify subjects by name.<br>Organizations that may inspect and/or copy research records maintained at the participating site for quality assurance and data analysis include groups such as the ethics committee, sponsor or the pertinent regulatory authorities.<br><br>**Benefit**<br><u>Benefits for an individual participant:</u><br>The benefit for the participant is a certainty in the course of his vaccination response as a result of the serological tests.<br><u>Benefits for other patients/sub groups/society:</u><br>The subgroup benefit lies in the determination of the real-life effectiveness of the various vaccines in patients with malignant pathologies either in “watch-and-wait” situations or under therapy with therapeutical agents probably affecting the immune system. This is of importance for the assessment of the further course of the pandemic from a public health point of view and from patient groups with malignant pathologies.<br><br>**Benefit-risk-assessment**<br>The potential benefits of the study, especially the benefits for the society but also for the individual participant, outweigh the potential risks. Only minimal study-related measures will be performed.<br>The administration of the vaccines and the resulting risks are independent of the participation in this trial.<br>Appropriate risk mitigation activities will be performed. Therefore, the overall benefit-risk-ratio is favorable.
**Trial Duration**	The study will last for 24 months.

**Total number of patients**	We plan to include 80 patients in this trial.
**Study population**	All patients will submit peripheral blood samples<br>a) prior (0-14 days) to receipt of the first dose of vaccination<br>b) (2)-8 weeks after receipt of the second dose<br>c) and 6,12 and 18 months following the first dose of vaccination (always +/- 2 weeks)
**Study visits**	**Visit 1:** prior (0-14 days) to first dose of vaccination<br>**Visit 2:** (2)-8 weeks after the second dose of vaccination<br>**Visit 3-5:** 6,12 and 18 months following the first dose of the vaccinations (always +/- 2 weeks)<br>The following intervention is planned at **each study visit**:<br>venous blood collection of max. 50ml blood (1-2 x 10ml serum; 3-4 x10ml EDTA blood)<br>Routine hematology and chemistry lab including hemogram, immunoglobuline-levels, lymphocyte subpopulations<br>The participation in the study will officially end after 18 months +/- 2 weeks after the first vaccination.

STROBE Statement—Checklist of items that should be included in reports of ***cohort studies***

	**Item No**	**Recommendation**	
**Title and abstract**	1	(*a*) Indicate the study's design with a commonly used term in the title or the abstract	✓
		(*b*) Provide in the abstract an informative and balanced summary of what was done and what was found	✓
**Introduction**			
Background/rationale	2	Explain the scientific background and rationale for the investigation being reported	✓
Objectives	3	State specific objectives, including any prespecified hypotheses	✓
**Methods**			
Study design	4	Present key elements of study design early in the paper	✓
Setting	5	Describe the setting, locations, and relevant dates, including periods of recruitment, exposure, follow-up, and data collection	✓
Participants	6	(*a*) Give the eligibility criteria, and the sources and methods of selection of participants. Describe methods of follow-up	✓
		(*b*) For matched studies, give matching criteria and number of exposed and unexposed	
Variables	7	Clearly define all outcomes, exposures, predictors, potential confounders, and effect modifiers. Give diagnostic criteria, if applicable	✓
Data sources/ measurement	8*	For each variable of interest, give sources of data and details of methods of assessment (measurement). Describe comparability of assessment methods if there is more than one group	
Bias	9	Describe any efforts to address potential sources of bias	na
Study size	10	Explain how the study size was arrived at	see also reporting summary
Quantitative variables	11	Explain how quantitative variables were handled in the analyses. If applicable, describe which groupings were chosen and why	na
Statistical methods	12	(*a*) Describe all statistical methods, including those used to control for confounding	✓
		(*b*) Describe any methods used to examine subgroups and interactions	na
		(*c*) Explain how missing data were addressed	✓
		(*d*) If applicable, explain how loss to follow-up was addressed	na
		(*e*) Describe any sensitivity analyses	na
**Results**			
Participants	13*	(a) Report numbers of individuals at each stage of study—eg numbers potentially eligible, examined for eligibility, confirmed eligible, included in the study, completing follow-up, and analysed	✓
		(b) Give reasons for non-participation at each stage	✓
		(c) Consider use of a flow diagram	
Descriptive data	14*	(a) Give characteristics of study participants (eg demographic, clinical, social) and information on exposures and potential confounders	✓
		(b) Indicate number of participants with missing data for each variable of interest	na
		(c) Summarise follow-up time (eg, average and total amount)	na
Outcome data	15*	Report numbers of outcome events or summary measures over time	na
Main results	16	(*a*) Give unadjusted estimates and, if applicable, confounder-adjusted estimates and their precision (eg, 95% confidence interval). Make clear which confounders were adjusted for and why they were included	✓
		(*b*) Report category boundaries when continuous variables were categorized	na
		(*c*) If relevant, consider translating estimates of relative risk into absolute risk for a meaningful time period	na

Other analyses	17	Report other analyses done—eg analyses of subgroups and interactions, and sensitivity analyses	na
**Discussion**			
Key results	18	Summarise key results with reference to study objectives	✓
Limitations	19	Discuss limitations of the study, taking into account sources of potential bias or imprecision. Discuss both direction and magnitude of any potential bias	✓
Interpretation	20	Give a cautious overall interpretation of results considering objectives, limitations, multiplicity of analyses, results from similar studies, and other relevant evidence	✓
Generalisability	21	Discuss the generalisability (external validity) of the study results	✓
**Other information**			
Funding	22	Give the source of funding and the role of the funders for the present study and, if applicable, for the original study on which the present article is based	✓

*Give information separately for exposed and unexposed groups.

Note: An Explanation and Elaboration article discusses each checklist item and gives methodological background and published examples of transparent reporting. The STROBE checklist is best used in conjunction with this article (freely available on the Web sites of PLoS Medicine at http://www.plosmedicine.org/, Annals of Internal Medicine at http://www.annals.org/, and Epidemiology at http://www.epidem.com/). Information on the STROBE Initiative is available at http://www.strobe-statement.org.
