# Supplementary material for: Potent high-avidity neutralizing antibodies and T cell responses after COVID-19 vaccination in individuals with B cell lymphoma and multiple myeloma
Source: Nat Cancer. 2022 Dec 21;4(1):81–95. doi: 10.1038/s43018-022-00502-x (PMC9886553; doi:10.1038/s43018-022-00502-x)
Supplement: Supplementary file 2 — Reporting Summary [file 43018_2022_502_MOESM2_ESM.pdf]

nature portfolio

Corresponding author(s): Oliver T. Keppler, M.D.

Last updated by author(s): 23rd of November 2022

Reporting Summary

Nature Portfolio wishes to improve the reproducibility of the work that we publish. This form provides structure for consistency and transparency in reporting. For further information on Nature Portfolio policies, see our Editorial Policies and the Editorial Policy Checklist.

Statistics

For all statistical analyses, confirm that the following items are present in the figure legend, table legend, main text, or Methods section.

n/a	Confirmed	
☐	☒	The exact sample size (*n*) for each experimental group/condition, given as a discrete number and unit of measurement
☒	☐	A statement on whether measurements were taken from distinct samples or whether the same sample was measured repeatedly
☐	☒	The statistical test(s) used AND whether they are one- or two-sided<br>*Only common tests should be described solely by name; describe more complex techniques in the Methods section.*
☒	☐	A description of all covariates tested
☐	☒	A description of any assumptions or corrections, such as tests of normality and adjustment for multiple comparisons
☐	☒	A full description of the statistical parameters including central tendency (e.g. means) or other basic estimates (e.g. regression coefficient) AND variation (e.g. standard deviation) or associated estimates of uncertainty (e.g. confidence intervals)
☐	☒	For null hypothesis testing, the test statistic (e.g. *F*, *t*, *r*) with confidence intervals, effect sizes, degrees of freedom and *P* value noted<br>*Give P values as exact values whenever suitable.*
☒	☐	For Bayesian analysis, information on the choice of priors and Markov chain Monte Carlo settings
☒	☐	For hierarchical and complex designs, identification of the appropriate level for tests and full reporting of outcomes
☐	☒	Estimates of effect sizes (e.g. Cohen's *d*, Pearson's *r*), indicating how they were calculated

Our web collection on statistics for biologists contains articles on many of the points above.

Software and code

Policy information about availability of computer code

Data collection	No software was used for data collection.
Data analysis	Data was analyzed using Prism 9.3.1. (GraphPad Software, USA).

For manuscripts utilizing custom algorithms or software that are central to the research but not yet described in published literature, software must be made available to editors and reviewers. We strongly encourage code deposition in a community repository (e.g. GitHub). See the Nature Portfolio guidelines for submitting code & software for further information.

Data

Policy information about availability of data

All manuscripts must include a data availability statement. This statement should provide the following information, where applicable:

- Accession codes, unique identifiers, or web links for publicly available datasets
- A description of any restrictions on data availability
- For clinical datasets or third party data, please ensure that the statement adheres to our policy

Pseudonymized participant data, including patient record data and all primary data from measurements conducted, are available in a public repository (DOI: 10.17632/z6dw96y8sw.1). The source data for all figures and extended data figures are available in the supplementary information. The SARS-CoV-2 sequences and protein data are available under accession codes PDB ID 6VXX, GenBank IDs MW717675.1 and MZ945494, and GISAID IDs EPI_ISL_412971, EPI_ISL_2557176, and EPI_ISL_8768822.2. Written informed consent to the publication of pseudonymized data has been been obtained from study participants. All other data supporting the findings of this study are available from the corresponding author on reasonable request.

Human research participants

Policy information about studies involving human research participants and Sex and Gender in Research.

Reporting on sex and gender	The sex is described in Table 1 of the manuscript, and in the pseudonymized participant data table (DOI: 10.17632/z6dw96y8sw.1).
Population characteristics	All patients aged 18 years or older and had a confirmed diagnosis of B-cell-Lymphoma or Multiple Myeloma were eligible.<br>The age, sex, disease remission status and treatment history are described in Table 1 of the manuscript , and in the pseudonymized participant data table (DOI: 10.17632/z6dw96y8sw.1).<br>All participants provided a written consent to participation in this study or to sample contribution to the Biobank FREEZE including the agreement to deposition of pseudonymized data.
Recruitment	All patients were recruited while visiting the outpatient center of the Medical Center – University of Freiburg, Faculty of Medicine, University of Freiburg, Germany.
Ethics oversight	Cancer patients: approval by the local Ethics Committee (21-1386) of the University of Freiburg, Freiburg, Germany.<br>Healthy individuals: approval by the local ethics committee (ethics vote 476/20 and 26/21S-SR) of the Technical University of Munich, German. The study is conducted according to the ethical principles of the Declaration of Helsiniki, Good Clinical Practice and applicable regulatory requirements.

Note that full information on the approval of the study protocol must also be provided in the manuscript.

Field-specific reporting

Please select the one below that is the best fit for your research. If you are not sure, read the appropriate sections before making your selection.

☒ Life sciences ☐ Behavioural & social sciences ☐ Ecological, evolutionary & environmental sciences

For a reference copy of the document with all sections, see nature.com/documents/nr-reporting-summary-flat.pdf

Life sciences study design

All studies must disclose on these points even when the disclosure is negative.

Sample size	No sample-size calculation was performed. When we started our sample collection no prior published data on COVID-19 vaccine response in cancer patients was available.<br>During the recruiting period all patients with either a B-cell lymphoma or multiple myeloma were invited to participate in the study, regardless of sex, age,and comorbidities. For accurate experimental analyses we aimed to receive patient material from before the first vaccination. After the recruiting period all patients in our outpatient center wanting to participate in the study were already vaccinated against COVID-19 and therefore no longer eligible for being included into our study.
Data exclusions	Patients were excluded, of whom no samples sufficient for evaluation could be collected. All serum samples were characterized for the prescence of anti-SARS-CoV2 anti-nucleocapsid antibodies.<br>In case of a positive anti-nucleocapsid titer, patients were excluded from data analysis (except for breakthrough infections).
Replication	The assays to determine binding antibody units (BAU) were performed using commercial, diagnostic well-validated tests that make use of calibrators, negative and positive controls. Titers were determined according to WHO standards BAU assuring high standardization. Antibody avidity was characterized using an established modified commercial assay that was validated previously showing low variance between results (Wratil, Stern, Priller et al., Nature Med 2022). The neutralization assay was validated previously (Wratil, Stern, Priller et al., Nature Med 2022) showing low variance between results of independent experiments. Each sample was tested in the neutralization assay at six different concentrations. Due to low sample volumes available, experiments to determine antibody concentration, antibody avidity and neutralization

	were not repeated. The counts of cell types and cell subsets and peripheral blood were determined using accredited diagnostics for patient samples showing high validation and standardization.<br>For IFN-γ ELISPOT assays, each sample was tested in three replicate wells for each antigen (peptide pool) within the same experiment. Sufficient samples for replication in technically independent IFN-γ ELISPOT tests were not available. After automated spot identification by the software, each well was visually inspected, and obvious artifacts (shadows generated by the ELISPOT plate structure or amorphous particles) were manually removed. Results for the three replicate wells for each sample and antigen were averaged. T-cell responses expressed as SFU were normalized to the frequency of T-cells and the individual PBMC samples. Normalization was mandatory because PBMCs is collected from the different hematologic patient groups showed heterogeneous T-cell abundance due to the high B-cell lymphocytosis characteristic of certain lymphomas ,e.g. chronic lymphocytic leukemia.<br>All attempts at replication were successful.
Randomization	Due to the fact that this was a longitudinal, observational cohort study, no randomization was performed. The intervention of interest in this study is SARS-CoV-2 vaccination. Participants were vaccinated by their primary care physicians or official vaccination centers. Timing of vaccination was independent of the study. Usually, patients were vaccinated in the time interval proposed by national authorities at the time.
Blinding	All laboratory assays were performed in blinded fashion. De-blinding of cohorts was performed after the evaluation of all raw data.

Reporting for specific materials, systems and methods

We require information from authors about some types of materials, experimental systems and methods used in many studies. Here, indicate whether each material, system or method listed is relevant to your study. If you are not sure if a list item applies to your research, read the appropriate section before selecting a response.

Materials & experimental systems

n/a	Involved in the study	
☐	☒	Antibodies
☐	☒	Eukaryotic cell lines
☒	☐	Palaeontology and archaeology
☒	☐	Animals and other organisms
☐	☒	Clinical data
☒	☐	Dual use research of concern

Methods

n/a	Involved in the study	
☒	☐	ChIP-seq
☒	☐	Flow cytometry
☒	☐	MRI-based neuroimaging

Antibodies

Antibodies used	Patients were not treated with antibodies as part of this study. Data on suppliers, catalog numbers, clone names and lot numbers of therapeutic antibodies that patients received before being included in the study are not available. The following commercially available kits that contain secondary antibodies were used to determine SARS-CoV-2 specific antibody responses: IgG agile SARS-CoV-2 ELISA (Virion/Serion, Germany, Cat.-No.: ESR400G), SARS-CoV-2 IgG II quant (Abbott, USA, Cat.-No.: 6S60).
Validation	Certified antibody assays were validated according to the manufacturers' instructions using positive and negative controls as well as calibrators.

Eukaryotic cell lines

Policy information about cell lines and Sex and Gender in Research

Cell line source(s)	MDA-MB-231 (German collection of Microorganisms and Cell Cultures, Germany), Vero-E6<br>(American Type Culture Collection, USA)
Authentication	Cells were authenticated by short tandem repeat (STR) analysis.
Mycoplasma contamination	Lines were regularly screened for mycoplasma contimination. No contaminations were detected.
Commonly misidentified lines<br>(See ICLAC register)	No commonly misidentified cell lines (according to ICLAC register) were used.

Clinical data

Policy information about clinical studies

All manuscripts should comply with the ICMJEguidelines for publication of clinical research and a completedCONSORT checklist must be included with all submissions.

Clinical trial registration	Cancer Patients: observational study; the local Ethics Committee (21-1386) of the University of Freiburg; registered at the Paul-Ehrlich Institute (NIS599) and Deutsches Register Klinischer Studien (DRKS00025901).<br>Healthy individuals: Ethics protocol of follow -up studies are: 476/20; 26/21S-SR; 229/21; no clinical trial was performed.
Study protocol	The study protocol synopsis from June 2021 (in English language) is provided in the Supplementary Information. Additionally, all other documents are available upon request to "andrea.hafkemeyer@uniklinik-freiburg.de".
Data collection	Data from patients were recruited into the study from March to May 2021 at the Freiburg University Medical Center.<br>The last blood sample included in this report was drawn on January 20, 2022. Clinical presentation of cancer patients was documented from the very beginning of the study until July 25th 2022.
Outcomes	The aim of our observational study was to extensively examine the humoral and cellular immune responses of this cancer patient cohort relative to a cohort of sex- and age-matched healthy individuals
